# *FGF* gene family characterization provides insights into its adaptive evolution in Carnivora

**DOI:** 10.1002/ece3.7814

**Published:** 2021-06-29

**Authors:** Qinguo Wei, Yuehuan Dong, Guolei Sun, Xibao Wang, Xiaoyang Wu, Xiaodong Gao, Weilai Sha, Guang Yang, Honghai Zhang

**Affiliations:** ^1^ Jiangsu Key Laboratory for Biodiversity and Biotechnology College of Life Sciences Nanjing Normal University Nanjing China; ^2^ College of Life Sciences Qufu Normal University Qufu China

**Keywords:** Carnivora, evolutionary pattern, fibroblast growth factors, positive selection

## Abstract

Fibroblast growth factors (FGFs) encoded by the *FGF* gene family can regulate development and physiology in animals. However, their evolutionary characteristics in Carnivora are largely unknown. In this study, we identified 660 sequences of three types of *FGF* genes from 30 unannotated genomes of Carnivora animals (before 7th May 2020), and the *FGF* genes from 52 Carnivora species were analyzed through the method of comparative genomics. Phylogenetic and selective pressure analyses were carried out based on the *FGF* genes of these 52 Carnivora species. The phylogenetic analysis results demonstrated that the *FGF* gene family was divided into 10 subfamilies and that *FGF5* formed one clade rather than belonging to the subfamilies of *FGF4* and *FGF6*. The evolutionary analysis results showed that the *FGF* genes were prominently subjected to purifying selection and were highly conserved in the process of Carnivora evolution. We also carried out phylogenetic comparative analyses, which indicated that the habitat was one of the factors that shaped the evolution of Carnivora *FGF* genes. The *FGF1* and *FGF6* genes were positively selected in the Carnivora animals, and positive selection signals were detected for the *FGF19* gene in semiaquatic Carnivora animals. In summary, we clarified the phylogenetic and evolutionary characteristics of Carnivora *FGF* genes and provided valuable data for future studies on evolutionary characterization of Carnivora animals.

## Graphical abstract

We identified 660 new sequences of three types of FGFs genes from 30 Carnivora animals’ unannotated genomes, and 1,144 FGFs genes from 52 Carnivora species were analyzed through the method of comparative genomics. The phylogenetic analysis clarified the classification of Carnivora FGF gene family. We found that FGFs genes mainly undergone purifying selection and the positively selected signals were found in FGF1, FGF6, and FGF19, which might be associated with the adaption of these animals for their diet habit and predation style. We also found that the habitat is one of the factors that shaped the evolution of Carnivora FGFs genes.

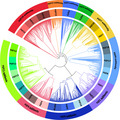

## 1 INTRODUCTION

Fibroblast growth factors (FGFs), which participate in diverse biological activities, such as lipid and carbohydrate metabolism, play vital roles in animal development and physiology. They are a family of very conservative growth factors and comprise 22 members in mammals (Brewer et al., 2016). The 22 FGFs can be divided into three types: the canonical FGFs (cFGFs), the hormone‐like FGFs (hFGFs), and the intracellular FGFs (iFGFs) (Itoh & Ornitz, 2011). While cFGFs and hFGFs are ligands that act through binding to appropriate receptors, iFGFs can act independently of receptor‐binding. The hFGFs contained members of FGF19, FGF21, and FGF23; the iFGFs include FGF11, FGF12, FGF13, and FGF14; the rest members (FGF1 to FGF10, FGF16, FGF 17, FGF18, FGF20, and FGF22) constitute the cFGFs (Ornitz & Itoh, 2015). The cFGFs play important roles in cell growth, differentiation, and organ development and formation, whereas hFGFs largely function as an endocrine factor; iFGF act as intracellular factors (Itoh & Ornitz, 2011). The *FGF* gene family was divided into several subfamilies based on phylogenetic relationships among these members. The hFGFs and iFGFs constitute one subfamily each. The cFGFs were divided into several other subfamilies while the number of subfamilies and the phylogenetic position of *FGF3* and *FGF5* remain ambiguous (Popovici et al., 2005).

The FGF family plays key roles in the development of animals and thus has attracted much attention in recent years (Imamura, 2014; Ornitz & Itoh, 2015). While FGF3 plays an important role in ear and tooth development (Itoh & Ornitz, 2008), FGF10 and FGF20 have vital roles in lung and kidney development (Barak et al., 2012). FGF5, FGF7, FGF10, FGF18, and FGF22 are involved in hair growth regulation (Imamura, 2014), and mutations in FGF9 can lead to the fusion of the elbow and knee joints in humans and murine animals (Harada et al., 2009; Wang et al., 2012). Further, FGF19 and FGF21 regulate energy homeostasis and thermogenesis (Imamura, 2014), while FGF23 is involved in the regulation of bone mineral density (Bhattacharyya et al., 2012). Additionally, iFGFs are involved in adaptation to hypoxia (Yang et al., 2015).

Carnivora, an order of mammals that largely feed on meat, with diverse habitats and feeding ecology, is one of the most species‐rich orders in mammals and is distributed widely across the world (Bekoff et al., 1984; Savage, 1977). The order Carnivora contains more than 200 species, which have great differences in morphology, ecology, and diet (Van Valkenburgh & Wayne, 2010). The body weight and size of Carnivora vary to a large extent, ranging from the least weasel (*Mustela nivalis*) weighing about 30 g to the male northern elephant seal weighing 2,300 kg (King, 1983; Smith & Xie, 2013; Van Valkenburgh & Wayne, 2010). The style of locomotion and habitat of Carnivora are also diverse and include semiaquatic swimmers (pinnipeds and lutrinae), climbers (martes), and diggers (melinae) (Barnes et al., 2008; Botton‐Divet et al., 2018; Wei et al., 2020). Besides these, the Carnivora animals have some characteristics in common, for example, relatively dense fur, excellent vision, hearing, and sense of smell (Van Valkenburgh & Wayne, 2010). The wide variation in the characteristics of Carnivora makes it an excellent order for studying the varied evolutionary scenarios that may have occurred over time. Since FGFs play important roles in the process of life development and maintenance, it is possible that there is a relationship between the diversity phenotype of Carnivora and the evolutionary characteristics of *FGF* genes. However, the evolutionary characteristics of *FGF* genes in Carnivora were still largely unknown until recently, and little is known about the relationship between the diversity phenotype of Carnivora and the evolutionary characteristics of *FGF* genes. Hence, it remains to be determined whether there are certain characteristics of *FGF* genes in this widely diverse animal order that are associated with the diversity phenotype described above. To address this question, we performed a comparative genomic study to illustrate the evolutionary characteristics of the *FGF* gene family in Carnivora animals and to probe into its relationship with the diversity phenotype of Carnivora animals.

## 2 MATERIALS AND METHODS

### 2.1 Genome data

**FIGURE 1:**
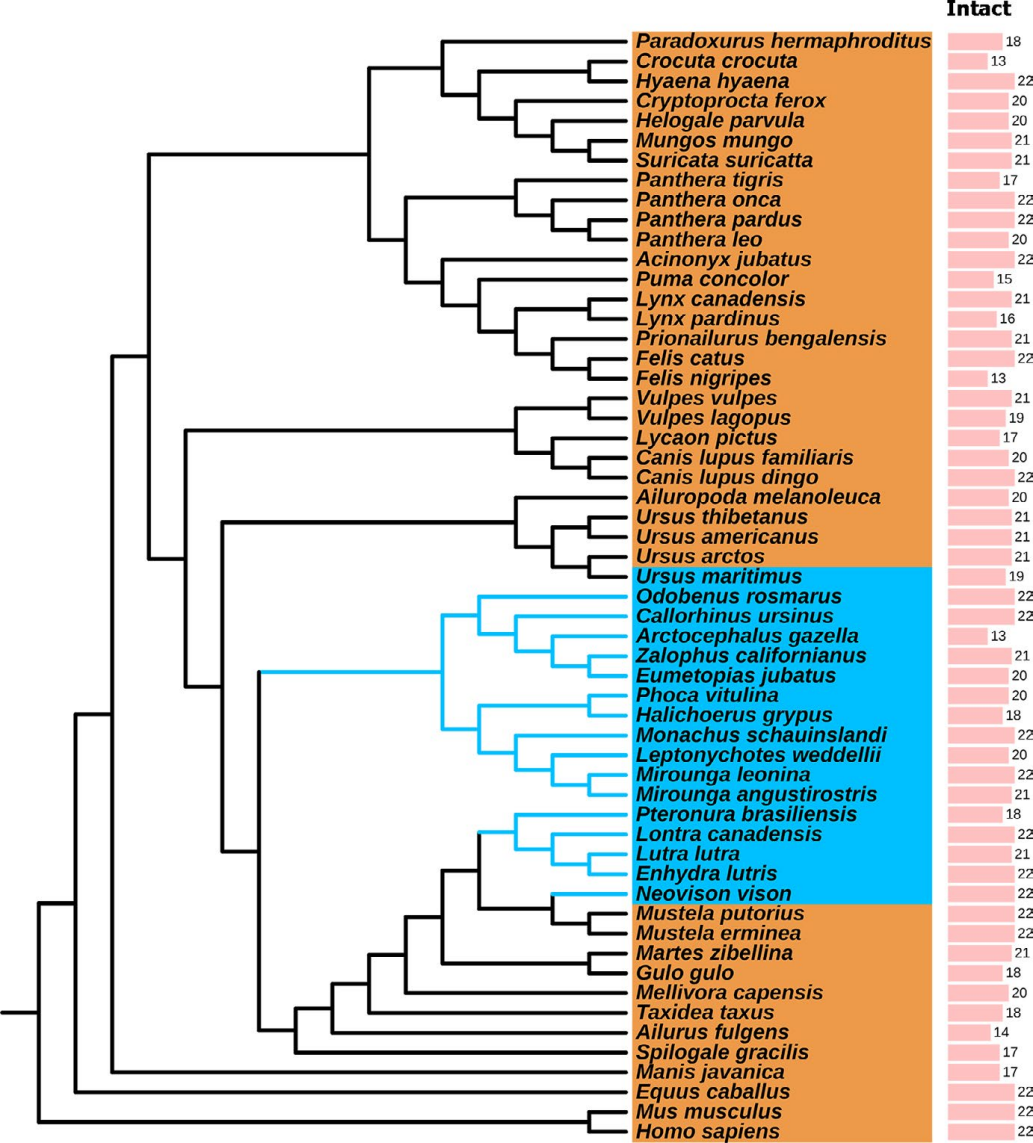
Species tree for the animals used in this study and the intact *FGF* gene number in these animals. The tree was downloaded from TimeTree (http://www.timetree.org/) and modified using Itol (https://itol.embl.de/). The blue clade indicates semiaquatic animals, and the orange clade represents terrestrial animals

Carnivora comprises two suborders: Caniformia and Feliformia. There were 52 Carnivora species genome sequences available in GenBank until 7 May 2020, including those for *Canis lupus dingo*, *Canis lupus familiaris*, *Vulpes vulpes*, *Vulpes lagopus*, and *Lycaon pictus* in the family Canidae; *Enhydra lutris kenyoni*, *Pteronura brasiliensis*, *Neovison vison*, *Mustela putorius furo*, *Gulo gulo*, *Mellivora capensis*, *Taxidea taxus jeffersonii*, *Mustela ermine*, *Lutra lutra*, *Lontra Canadensis*, and *Martes zibellina* in the family Mustelidae; *Ailurus fulgens styani* in the family Ailuridae; *Spilogale gracilis* in the family Mephitidae; *Zalophus californianus*, *Eumetopias jubatus*, *Callorhinus ursinus*, and *Arctocephalus gazelle* in the family Otariidae; *Odobenus rosmarus divergens* in the family Odobenidae; *Leptonychotes weddellii*, *Neomonachus schauinslandi*, *Phoca vitulina*, *Mirounga angustirostris*, and *Mirounga leonine* in the family Phocidae; *Ursus arctos horribilis*, *Ursus maritimus*, *Ursus americanus*, *Ailuropoda melanoleuca*, *Ailuropoda melanoleuca*, and *Ursus thibetanus* in the family Ursidae; *Panthera tigris altaica*, *Felis nigripes*, *Panthera onca*, *Acinonyx jubatus*, *Puma concolor*, *Panthera pardus*, *Felis catus*, *Lynx pardinus*, *Prionailurus bengalensis*, *Panthera leo,* and *Lynx canadensis* in the family Felidae; *Hyaena hyaena* and *Crocuta crocuta in* the family Hyaenidae; *Suricata suricatta*, *Helogale parvula*, and *Mungos mungo* in the family Herpestidae; *Cryptoprocta ferox* in the family Eupleridae; *Paradoxurus hermaphrodites* in the family Viverridae. Among these, there were 30 unannotated genomes, which were downloaded from the NCBI Genome database for *FGF* gene identification. The information of genomes used in this study is listed in Table S1. The Carnivora animals used in this study were classified into terrestrial and semiaquatic groups according to their lifestyle (Figure 1, the blue clade indicates semiaquatic animals, and the orange clade represents terrestrial animals). The accession numbers of *FGF* genes downloaded from the GenBank database and the abbreviation of the species names are listed in Tables S2 and S3, respectively.

### 2.2 Identification of *FGF* genes

First, we retrieved *FGF* genes of humans, mice, domestic dogs, domestic cats and used them as queries. Next, we retrieved *FGF* genes from other species through local BLAST, selecting results under the E‐value of 1e–5. The retrieved sequences were classified into three types: the sequences containing a presumed start and stop codon were considered as intact genes; the sequences with premature stop codons or frame‐shifts were classified as pseudogenes; and sequences longer than 100 bp containing a start or a stop codon were classified as partial genes. The partial genes were assessed to determine whether they were from independent loci or not, and whether they were unique. Finally, all of the three types of genes were searched in the GenBank using BLASTP to verify all the candidate genes belonging to the *FGF* gene family. All of the verified *FGF* gene sequences are shown in Data S1 in database Dryad, https://doi.org/10.5061/dryad.02v6wwq39.

### 2.3 Phylogenetic analysis of *FGF* genes

Phylogenetic analysis was conducted to clarify the evolutionary history and relationships of *FGF* genes in Carnivora. Human and mouse *FGF* genes were selected as the outgroup to determine the homology of the newly obtained *FGF* genes in Carnivora. First, the *FGF* gene nucleotide sequences were aligned using MUSCLE (Edgar, 2010) and adjusted manually. To build the maximum likelihood (ML) phylogenetic tree, the best model was determined through the method of “Find the best model” program embedded in IQ‐TREE and the ML tree was subsequently built using the IQ‐TREE (Nguyen et al., 2015) with 1,000 ultrafast bootstrap replications and a GTR + F + R8 sequence evolution model. For the Bayesian inference (BI) tree, we used MrModeltest 2.4 (Nylander, 2004) to choose the best model and MrBayes 3.2.7a (Ronquist et al., 2012) to construct the BI tree with one cold and three heat Markov chains with 4 × 10^7^ generations.

### 2.4 Codon‐based analysis of positive selection

Intact coding sequences of *FGF* genes were aligned using the software PRANK (Löytynoja, 2014) following the codon model and were selected for codon‐based analysis of positive selection (Table S3). Only the intact genes were selected for this evolutionary analysis because we think that functional genes are important for life. Further, the partial genes and pseudogenes were more likely influenced by the Sequencing and annotation technology, and thus were not included in the following analysis. The CODEML program in PAML 4 (Yang, 2007) was used to test the selection pressures in the Carnivora *FGF* genes with the framework of ML. The guide tree was downloaded from TimeTree (http://www.timetree.org/). First, the branch model (free ratio) was used to test the overall evolutionary characteristics in all branches. Then, the nonsynonymous to synonymous substitution rate (dN/dS) ratios for terrestrial and semiaquatic animals were estimated separately with the branch model, and the two‐ratio model and one‐ratio model were compared to test whether there was a difference between them. Second, the site model was used to identify positive selection signatures from all branches (Yang et al., 2000). The selection model (M2) was compared with the null model (M1), and a likelihood‐ratio test was performed to test for statistical significance. Finally, the branch‐site model was used to test the evolutionary characteristics of terrestrial and semiaquatic animals respectively.

### 2.5 Phylogenetic comparative analyses

The correlation of the dN/dS ratios of the *FGF* gene family in the two‐ratio model between terrestrial and semiaquatic Carnivora was tested using the cor.test function in R software. The phylogenetic independent contrast (PIC) analysis method (Felsenstein, 1985) was then used to investigate the relationship between the dN/dS ratios and habitat type while controlling for phylogeny. The dN/dS ratios from the free‐ratio model results were selected for the PIC analysis (Table S4). *FGF3, FGF6, FGF19,* and *FGF21* were selected for PIC analysis as they had more than three valid dN/dS ratios for each group. The PIC analyses were performed using R software with ape packages (Orme et al., 2012).

## 3 RESULTS

### 3.1 *FGF* gene identification and gene tree reconstruction

**FIGURE 2:**
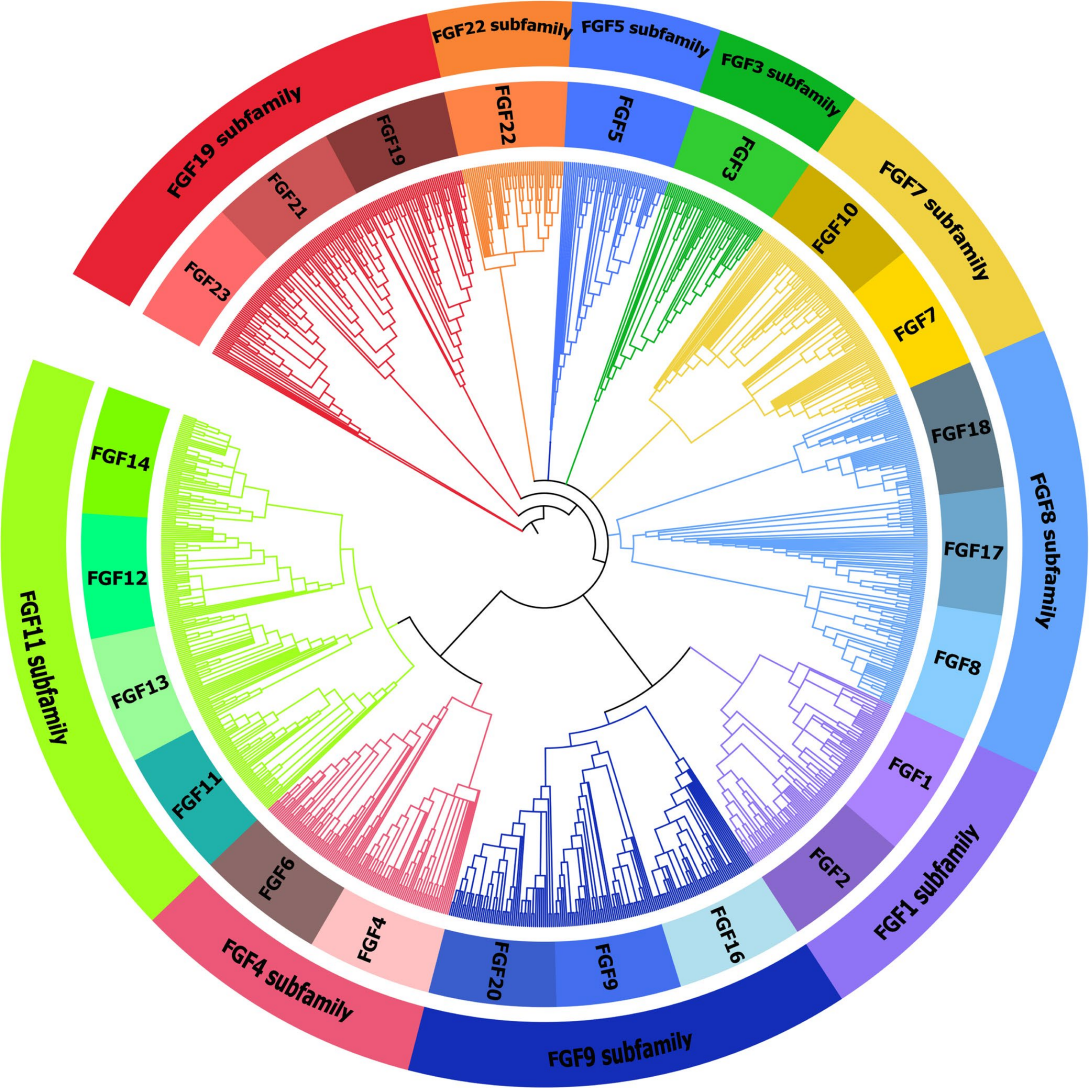
Phylogenetic tree of Carnivora *FGF* genes and classification of these genes according to Bayesian inference methods. The tree was constructed using MrBayes 3.2.7a and modified using Itol (https://itol.embl.de/)

A total of 660 new *FGF* genes were identified from 30 unannotated genomes, of which 566 were intact genes, 60 were partial genes, and 34 were pseudogenes (Data S1, Figure 1). All the species had 22 *FGF* genes, and the intact gene numbers in each species (Figure 1) were not correlated with the Genome contig N50 (Table S1), which validated the correction in the division of these *FGF* genes. The topologies of the ML tree (Figure S1) and BI tree (Figure S2) were similar, and both showed that the newly identified *FGF* genes were correctly classified into certain groups, validating the gene classification performed above. All these genes showed typical features of the *FGF* gene family, and the *FGF* gene subfamilies were clustered into a monophyletic group with high bootstrap values (Figure S1). The phylogenetic tree demonstrated that the Carnivora *FGF* genes were classified into 10 subfamilies (Figure 2): the *FGF1* subfamily (*FGF1, 2*), *FGF3* subfamily (*FGF3*), *FGF4* subfamily (*FGF4, 6*), *FGF5* subfamily (*FGF5*), *FGF7* subfamily (*FGF7, FGF10*), *FGF8* subfamily (*FGF8, FGF17, FGF18*), *FGF9* subfamily (*FGF9, FGF16, FGF20*), *FGF22* subfamily (*FGF22*), iFGF subfamily (*FGF11, FGF12, FGF13, FGF14*), and hFGF subfamily (*FGF19, FGF21, FGF23*).

### 3.2 Selection characteristics of *FGF* genes

The selection characteristics of *FGF* genes were analyzed using PAML 4 software based on the nonsynonymous to synonymous substitution rate ratio (*ω* = dN/dS). The selection type was determined by the *ω* values. The purifying selection, neutral selection, and positive selection were indicated with *ω* < 1, *ω* = 1, and *ω* > 1, respectively. The branch model (free‐ratio model) results indicated that the *FGF* genes mainly underwent purifying selection (Table S4). When we divided the Carnivora animals into terrestrial and semiaquatic groups, the two‐ratios and one‐ratio program were compared. The dN/dS ratios between these two groups were significantly different for the *FGF1, FGF6, FGF10, FGF11, FGF18*, *FGF19,* and *FGF21* genes (Table S5). We also found positive selection sites in *FGF1* and *FGF6* through the site model among all branches, which demonstrated that these two *FGF* genes were under positive selection (Table 1). Finally, we investigated the evolutionary characteristics of *FGF* genes in the terrestrial and semiaquatic groups respectively. The positive selection gene in the semiaquatic group was found to be *FGF19*, whereas no positively selected gene was detected in the terrestrial group (Table 2).

**TABLE 1:** Positive selection on Carnivora *FGF* genes through site model

Gene	Model										2ΔlnL	*p* Value	Positively selected sites (BEB)
	M1				M2								
	p0	p1	ω0	lnL	p0	p1	p2	ω0	ω2	lnL			
***FGF1***	**0.936**	**0.064**	**0.023**	**−1,065.370**	**0.939**	**0.033**	**0.028**	**0.027**	**3.456**	**−1,058.964**	**12.814**	.**002**	**57 I** **0.998** ** **58Q** **0.978** *
*FGF2*	1.000	0.000	0.024	−452.713	1.000	0.000	0.000	0.024	17.652	−452.713	0.000	1.000	
*FGF3*	0.959	0.041	0.029	−1717.299	0.959	0.041	0.000	0.029	22.761	−1717.299	0.000	1.000	
*FGF4*	0.993	0.007	0.026	−2007.588	0.993	0.007	0.000	0.026	31.071	−2007.588	0.000	1.000	
*FGF5*	0.579	0.421	0.082	−1512.133	0.636	0.000	0.364	0.120	1.471	−1,510.061	4.145	.126	
***FGF6***	**0.826**	**0.174**	**0.060**	**−1797.325**	**0.825**	**0.165**	**0.010**	**0.063**	**4.788**	**−1793.751**	**7.148**	.**028**	**13 Q** **0.985** *
*FGF7*	0.938	0.062	0.019	−1573.169	0.942	0.052	0.006	0.022	3.800	−1572.511	1.317	.518	
*FGF8*	1.000	0.000	0.004	−489.067	1.000	0.000	0.000	0.004	32.093	−489.067	0.001	1.000	
*FGF9*	0.988	0.012	0.002	−1,187.792	0.991	0.000	0.009	0.002	3.256	−1,186.162	3.259	.196	37 E 0.959*
*FGF10*	0.949	0.051	0.044	−1596.681	0.949	0.036	0.015	0.044	1.000	−1596.681	0.000	1.000	
*FGF11*	0.973	0.027	0.031	−1,263.881	0.973	0.027	0.000	0.031	17.839	−1,264.746	1.730	.421	
*FGF12*	1.000	0.000	0.008	−1,395.964	1.000	0.000	0.000	0.008	1.000	−1,395.963	0.002	.999	
*FGF13*	0.993	0.007	0.009	−1808.063	0.994	0.000	0.006	0.009	1.178	−1808.034	0.057	.972	
*FGF14*	0.981	0.019	0.009	−1,432.407	0.985	0.009	0.006	0.010	3.751	−1,430.178	4.458	.108	118 V 0.955*
*FGF16*	0.946	0.054	0.016	−1,062.600	0.946	0.054	0.000	0.016	9.683	−1,062.600	0.000	1.000	
*FGF17*	0.982	0.018	0.003	−1656.630	0.982	0.018	0.000	0.003	7.643	−1656.630	0.000	1.000	
*FGF18*	0.969	0.031	0.008	−1,029.257	0.969	0.016	0.016	0.008	1.000	−1,029.257	0.000	1.000	
*FGF19*	0.874	0.126	0.081	−2,464.904	0.873	0.119	0.008	0.082	4.275	−2,463.147	3.514	.173	
*FGF20*	0.961	0.039	0.017	−1,320.609	0.961	0.039	0.000	0.017	25.200	−1,320.609	0.000	1.000	
*FGF21*	0.742	0.258	0.086	−4,110.157	0.742	0.149	0.110	0.086	1.000	−4,110.157	0.000	1.000	
*FGF22*	0.909	0.091	0.038	−989.968	0.909	0.064	0.026	0.038	1.000	−989.968	0.000	1.000	
*FGF23*	0.969	0.031	0.060	−1,213.844	0.969	0.016	0.015	0.060	1.000	−1,213.844	0.000	1.000	

Notes

The bold values represent the positively selected genes.

* Means Bayes Empirical Bayes (BEB) > 0.95

** means BEB > 0.99.

**TABLE 2:** Positive selection on semiaquatic Carnivore animals’ *FGF* genes through branch‐site model

Gene name	Model					2ΔlnL	*p* Value	Positively selected sites (BEB)
	Null model		Alternative model					
	ω0	lnL	ω0	ω2	lnL			
*FGF1*	0.012	−2,297.611	0.012	1.000	−2,297.043	1.137	.286	
*FGF2*	0.000	−10,140.558	0.002	23.192	−10,132.951	15.215	<.001	
*FGF3*	0.040	−5,735.043	0.041	1.255	−5,734.911	0.263	.608	
*FGF4*	0.050	−3,742.312	0.050	1.000	−3,742.312	0.000	1.000	
*FGF5*	0.050	−7,459.411	0.050	3.255	−7,459.361	0.101	.750	
*FGF6*	0.048	−2,698.457	0.048	2.819	−2,697.668	1.578	.209	
*FGF7*	0.019	−1573.169	0.019	1.000	−1573.375	0.411	.521	
*FGF8*	0.000	−5,533.423	0.000	1.000	−5,533.423	0.000	.997	
*FGF9*	0.003	−2,220.490	0.003	1.000	−2,220.490	0.000	1.000	
*FGF10*	0.034	−2,743.907	0.034	1.000	−2,743.557	0.700	.403	
*FGF11*	0.031	−3,086.404	0.031	1.000	−3,086.404	0.000	.999	
*FGF12*	0.008	−2054.954	0.008	1.000	−2054.954	0.000	1.000	
*FGF13*	0.009	−3,227.924	0.009	7.132	−3,227.817	0.214	.643	
*FGF14*	0.012	−4,323.453	0.013	2.883	−4,323.403	0.101	.751	
*FGF16*	0.028	−2,862.934	0.028	1.730	−2,862.832	0.204	.652	
*FGF17*	0.007	−3,157.811	0.007	1.000	−3,157.811	0.000	1.000	
*FGF18*	0.003	−2,348.013	0.003	1.343	−2,347.923	0.179	.672	
***FGF19***	**0.080**	**−5,149.852**	**0.083**	**12.909**	**−5,139.542**	**20.621**	**<.001**	**366 I 0.996** ** **368 Y 1.000** **
*FGF20*	0.036	−3,536.128	0.035	1.000	−3,536.185	0.115	.735	
*FGF21*	0.075	−5,018.670	0.075	1.000	−5,018.670	0.000	1.000	
*FGF22*	0.075	−4,954.036	0.075	1.000	−4,954.036	0.000	.991	
*FGF23*	0.072	−5,709.082	0.072	1.000	−5,709.079	0.005	.941	

Notes

The bold values represent the positively selected genes.

** Means BEB > 0.99.

### 3.3 Correlation between dN/dS ratios and ecological factors

**FIGURE 3:**
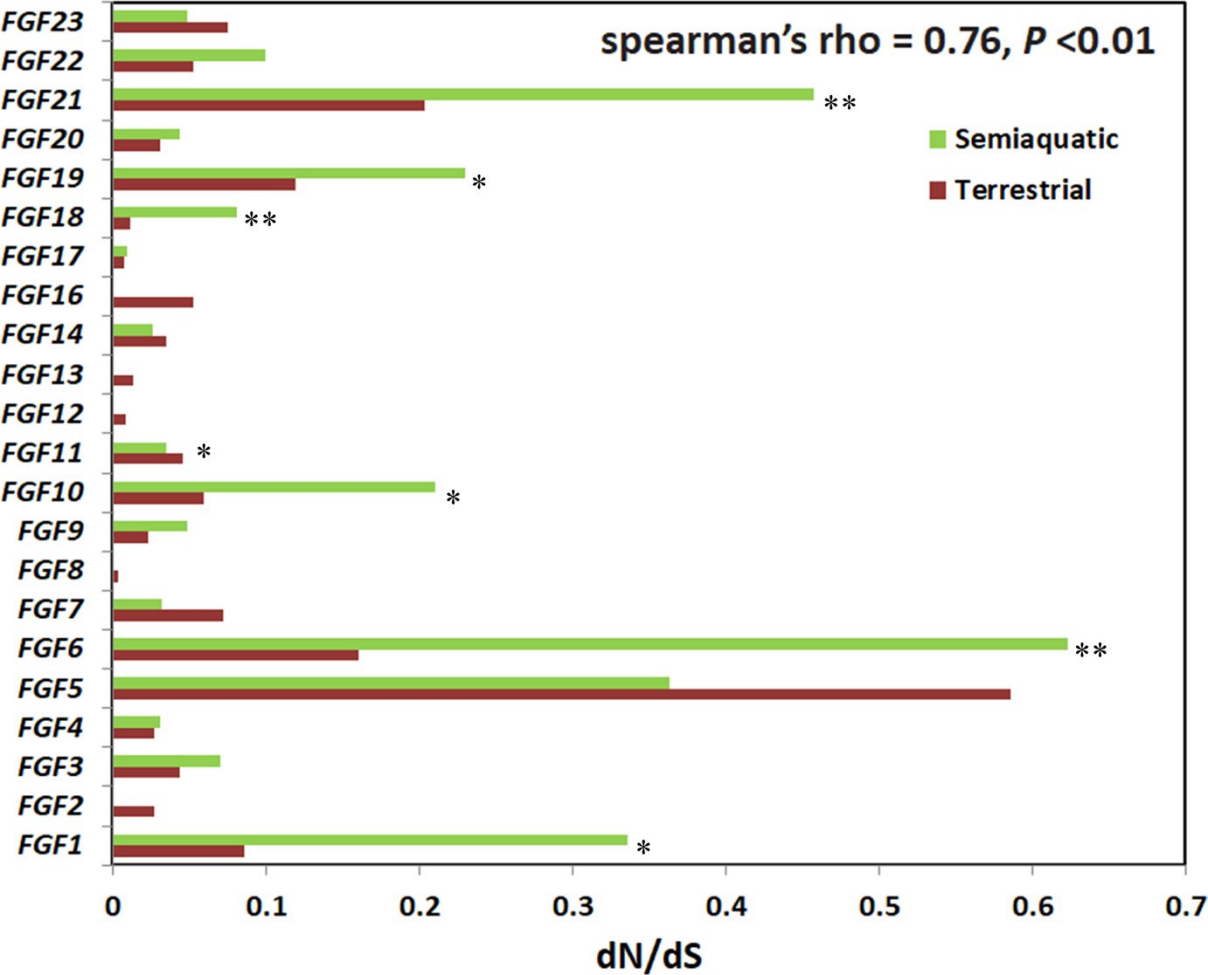
Comparison of dN/dS ratios between the terrestrial and semiaquatic group

**FIGURE 4:**
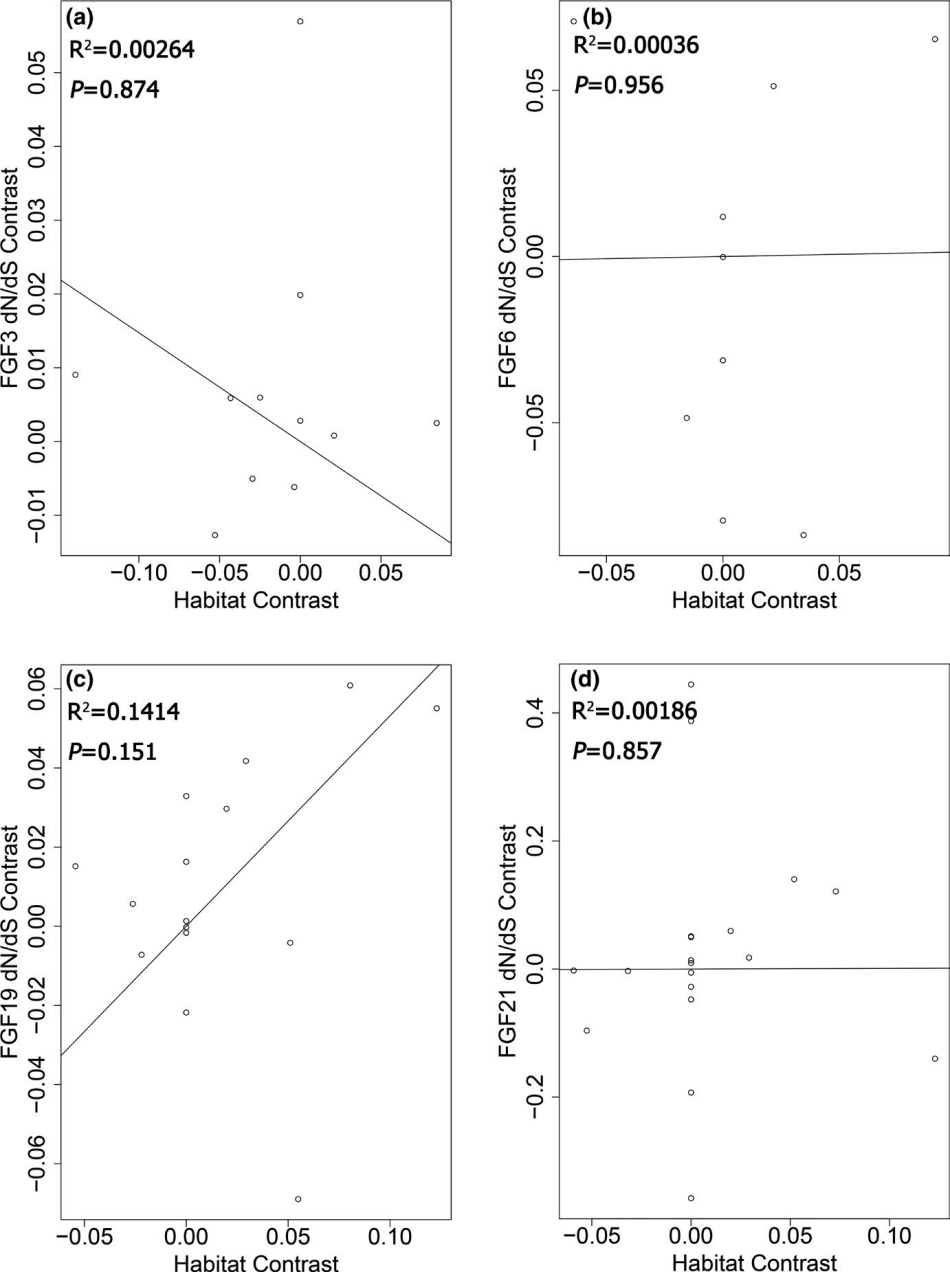
PIC analyses between the dN/dS ratios and habitat type in Carnivora animals

The correlation of the dN/dS ratios of the *FGF* gene family between the terrestrial and semiaquatic groups based on the two‐ratio model was significant (Spearman's rho = 0.7622549, *p* = .0005739) (Figure 3). The PIC results demonstrated that the dN/dS ratios for the *FGF3, FGF6, FGF9*, and *FGF21* genes were related to animal habitat type to a certain extent, and the difference did not reach to the significant level (Figure 4). Among these, the dN/dS ratios for *FGF19* demonstrated a stronger relationship with habitat than did those for the other three genes as it had the least *p* value (*p* = .151).

## 4 DISCUSSION

Carnivora is one of the most species‐rich orders in mammals, and its vast diversity in morphology, physiology, and ecological habit make it a suitable and widely‐studied group for evolutionary studies (Bekoff et al., 1984; Savage, 1977). The *FGF* gene family plays vital roles in animal development, but its evolutionary characteristics in Carnivora are still largely unknown. Further, it is still unclear whether there is a relationship between the evolutionary characteristics of *FGF* genes and the diversity phenotype of Carnivora. Hence, we explored the molecular adaptation of *FGF* genes during Carnivora evolution using comparative genomics.

To clarify the phylogenetic relationship of the Carnivora *FGF* gene family, we reconstructed the gene tree according to the ML and BI methods. According to the gene tree, the result of *FGF1* subfamily (*FGF1, 2*), *FGF3* subfamily (*FGF3*), *FGF8* subfamily (*FGF8, FGF17, FGF18*), *FGF9* subfamily (*FGF9, FGF16, FGF20*), iFGF subfamily (*FGF11, FGF12, FGF13, FGF14*), and hFGF subfamily (*FGF19, FGF21, FGF23*) was consistent with previous studies (Itoh & Ornitz, 2004; Oulion et al., 2012). The Carnivora *FGF5* genes were found to be closely related to *FGF9, FGF16*, and *FGF20*, according to the gene tree reconstructed through the ML method (Figure S1). However, the *FGF5* genes formed one separate clade according to the BI tree (Figure S2). In previous studies, the mammalian *FGF* gene family was classified into six or seven subfamilies (Itoh & Ornitz, 2004; Kim, 2001). In a study including protostomes, deuterostomes, and baculoviruses, the *FGF* gene family was divided into eight subfamilies (Popovici et al., 2005). Similarly, the *FGF* gene family had also been classified into eight subfamilies; wherein, the *FGF5* genes were placed in a subfamily comprising *FGF4, FGF5*, and *FGF6,* while *FGF3* was classified into one independent clade (Oulion et al., 2012). Additionally, *FGF* genes were classified into seven subfamilies in a recent study, which classified *FGF3* genes into a subfamily containing *FGF3*, *FGF7, FGF10*, and *FGF22*, while *FGF4, FGF5*, and *FGF6* were placed in one single subfamily (Zhang et al., 2019). However, *FGF5* was placed in *FGF1* subfamily according to synteny analyses (Itoh & Ornitz, 2008). In summary, the classification of *FGF* genes into eight subfamilies is widely accepted; however, the phylogenetic position of *FGF3* and *FGF5* remains ambiguous. For example, one study considered that *FGF3* and *FGF5* might each belong to an independent subfamily (Popovici et al., 2005). Both *FGF3* and *FGF5* were classified into one independent subfamily, based on the BI tree in this study (Figure 2). We predict that the classification of the *FGF* gene family may be influenced by the animal taxon based on the comprehensive analysis of our results and previous studies. Although most studies classified *FGF22* into the subfamily including *FGF3, FGF7, FGF10,* and *FGF22* or *FGF7, FGF10,* and *FGF22* (Itoh & Ornitz, 2004; Ornitz & Itoh, 2015; Oulion et al., 2012; Popovici et al., 2005), *FGF22* did not cluster with *FGF7* and *FGF10* into one clade according to the phylogenetic tree in our study (Figure 2). FGF22 has a putative N‐terminal signal peptide when compared with other cFGFs members (Itoh & Ornitz, 2004). Besides, *FGF22* also formed one clade according to the phylogenetic tree (Figure 2). Therefore, we propose that the Carnivora *FGF* gene family should be divided into 10 subfamilies, with *FGF3*, *FGF5*, and *FGF22* forming one subfamily each.

The Carnivora *FGF* genes mainly underwent purifying selection (Tables S4 and S5) during the evolution of Carnivora animals, and this was reflected in their conservative function during the development of animals (Itoh & Ornitz, 2004). The site model showed that the *FGF1* and *FGF6* genes were under positive selection in Carnivora (Table 1). Previous studies have demonstrated that *FGF1* functionally mediates pancreatic islet insulin secretion and modulates pancreatic β‐cell functions, which can maintain normal glucose levels (Gasser et al., 2017; Kolodziejski et al., 2020; Tennant et al., 2019). *FGF1* also plays vital roles in lipid metabolism through the FGF1/FGFR1 signaling pathway and may aid in obesity prevention (Wang et al., 2020). The main diet of Carnivora animals is meat, which is a hypercaloric food. Therefore, the positively selected *FGF1* gene in Carnivora may play an important role in adaptation to their hypercaloric diet. *FGF6* is regarded as an important factor that functions in muscle generation, differentiation, regeneration, integrity, and protection against mechanical stress (Armand et al., 2005; Laziz et al., 2007). *FGF6* also plays key roles in osteogenesis and regulation of bone metabolism through its activity on osteoclasts and osteoblasts (Bosetti et al., 2010). Carnivora animals have strong skeletal muscular systems to adapt to their predatory styles. The positive selection signal we found in the Carnivora *FGF6* gene might reflect the important role of this gene in the evolution of Carnivora animals and the maintenance of their strong skeletal muscular system. The positive selection signal was also found in *FGF19* in the semiaquatic Carnivora group through the branch‐site model (Table 2). The *FGF19* gene is an ileum‐derived key molecular mediator that acts on several metabolic processes, including the regulation of bile acid, lipid, and glucose metabolism homeostasis (Katarzyna et al., 2019; Lan et al., 2017). Therefore, *FGF19* plays important roles in postprandial metabolism and maintains the balance of animal shape and thermogenesis (Antonellis et al., 2019; Kir et al., 2011). Life in an aquatic habitat requires high energy consumption for thermoregulation by aquatic and semiaquatic animals living in this habitat, as water can conduct heat much more effectively than air (Schmidt‐Nielsen, 1997; Williams et al., 1998). The positive selection signal we found in *FGF19* may indicate that it plays vital roles in thermoregulation and weight balance in semiaquatic Carnivora animals.

The relationship of dN/dS ratios of *FGF* genes between the terrestrial and semiaquatic groups indicated that the habitat type had shaped the evolution process of the *FGF* genes (Figure 3, Table S5). This finding is consistent with a previous study that focused on the *FGF* gene family with regard to aquatic adaptation in cetaceans (Nam et al., 2017). We report that most of the *FGF* genes in the semiaquatic group had higher dN/dS ratios than those in the terrestrial group (Figure 3). We inferred that these genes may have undergone accelerated evolution during the evolution of these semiaquatic animals. Using the PIC analysis method, we found that among *FGF3, FGF6, FGF19,* and *FGF21*, *FGF19* had a stronger relationship with habitat type, which might be attributed to the higher energy metabolism requirement of semiaquatic Carnivora animals.

In summary, we identified 660 new *FGF* gene sequences in the order Carnivora and analyzed the evolutionary characteristics of the Carnivora *FGF* gene family. Based on the results of this study, we propose that the Carnivora *FGF* gene family should be classified into 10 subfamilies. Positive selection signals were found in *FGF1* and *FGF6*, which are functionally involved in glycometabolism and muscle development, respectively, indicating that these genes play important roles in Carnivora animals for their diet and predatory habits. The positive selection signals found in *FGF19* in the semiaquatic group demonstrated that *FGF19* plays a vital role in adaptation of animals to a semiaquatic lifestyle. Furthermore, we also found that the habitat type shaped the evolution of *FGF* genes in the order Carnivora. Thus, our findings provide important basis for future evolutionary studies on Carnivora animals.

## CONFLICT OF INTEREST

The authors declare that they have no competing interests.

## AUTHOR CONTRIBUTION

**Qinguo Wei:** Data curation (lead); Formal analysis (lead); Writing‐original draft (lead); Writing‐review & editing (lead). **Yuehuan Dong:** Data curation (equal). **Guolei Sun:** Data curation (equal); Formal analysis (equal). **Xibao Wang:** Data curation (equal). **Xiaoyang Wu:** Data curation (equal); Formal analysis (equal). **Xiaodong Gao:** Data curation (equal). **Weilai Sha:** Supervision (equal). **Guang Yang:** Conceptualization (equal); Project administration (equal). **Honghai Zhang:** Conceptualization (lead); Project administration (lead); Supervision (lead).

### DATA AVAILABILITY STATEMENT

All the genomes and annotated *FGF* gene sequences used in this study were accessed through the GenBank database using the accession numbers in Table S1 and S2. Both the annotated DNA sequence and DNA sequence identified from the genomes used for analysis in this study are provided in Data S1 (https://doi.org/10.5061/dryad.02v6wwq39).

## Supporting information

Fig S1

Fig S2

Table S1

Table S2

Table S3

Table S4

Table S5
