# Supplementary figures and images for: FGF gene family characterization provides insights into its adaptive evolution in Carnivora
Source: Ecol Evol. 2021 Jun 29;11(14):9837–47. doi: 10.1002/ece3.7814 (PMC8293770; doi:10.1002/ece3.7814)

Tree scale: 10

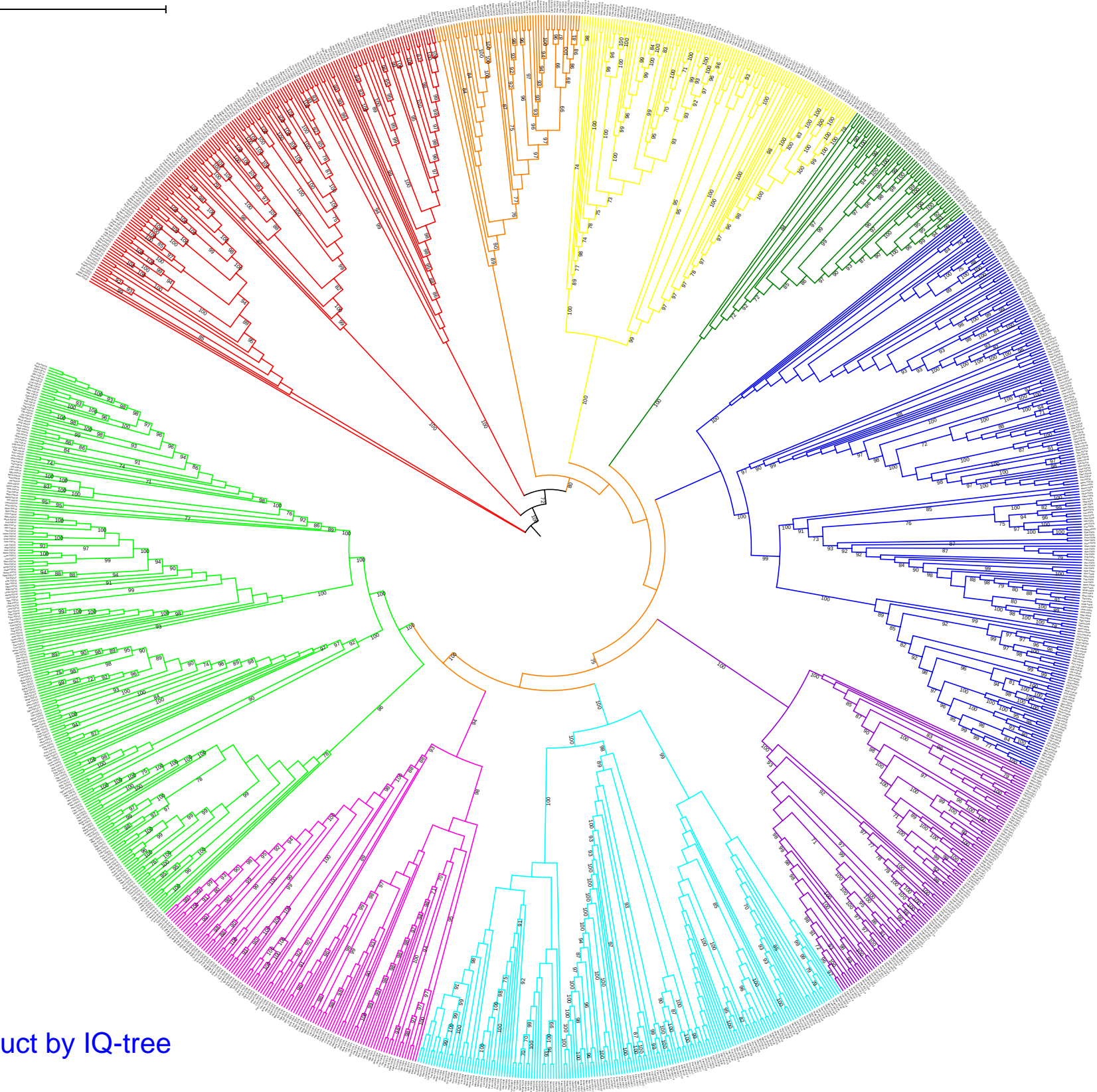

Figure S1 The FGF genes ML tree reconstruct by IQ-tree

Supplement: Supplementary file 1 — Fig S1 [file ECE3-11-9837-s006.pdf]

Tree scale: 0.1

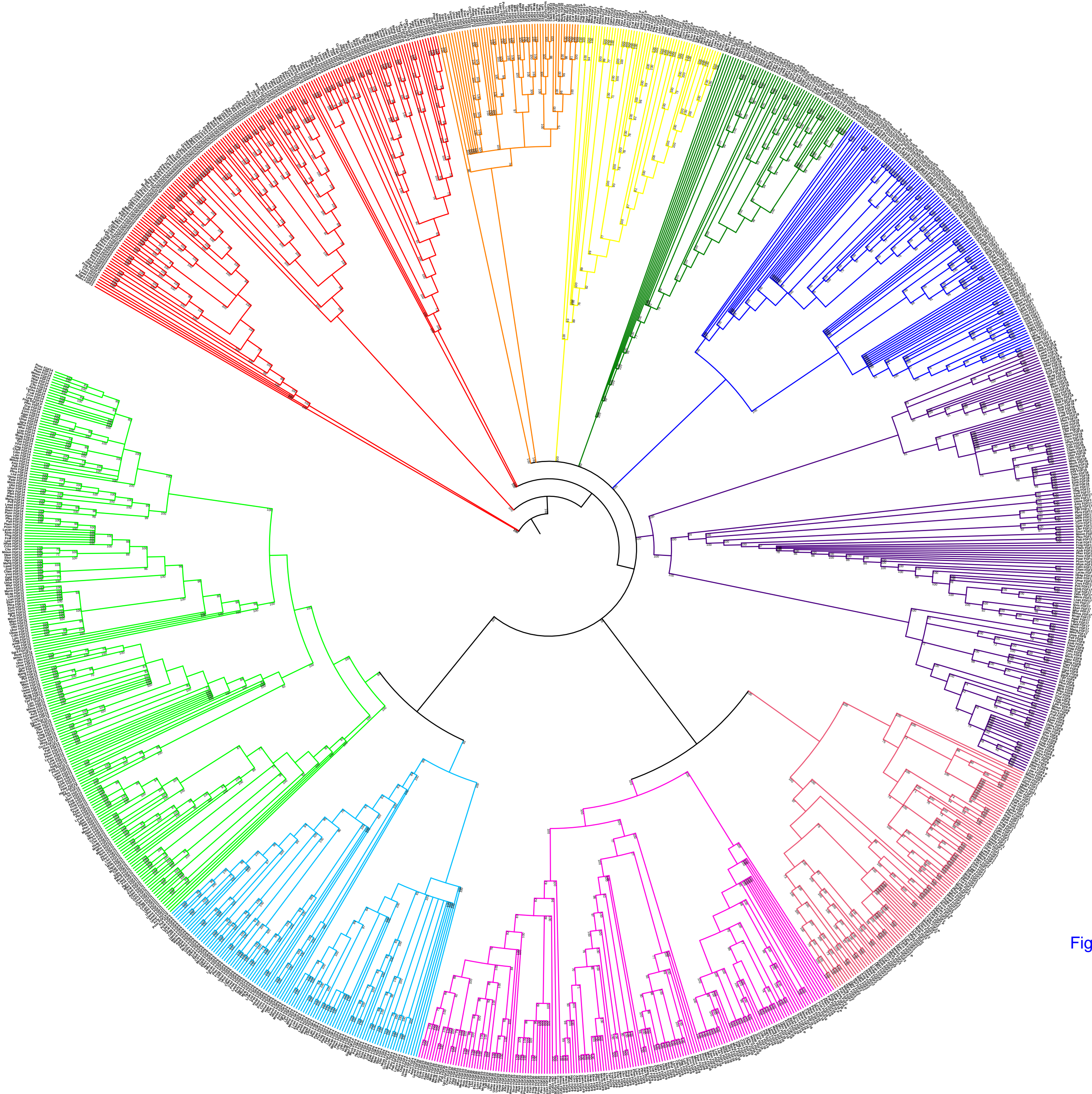

Figure S2 The FGF genes BI tree reconstructed by MrBayes

Supplement: Supplementary file 2 — Fig S2 [file ECE3-11-9837-s003.pdf]
