# Supplementary material for: FGF gene family characterization provides insights into its adaptive evolution in Carnivora
Source: Ecol Evol. 2021 Jun 29;11(14):9837–47. doi: 10.1002/ece3.7814 (PMC8293770; doi:10.1002/ece3.7814)
Supplement: Supplementary file 3 — Table S1 [file ECE3-11-9837-s005.pdf]

Table S1 Genomic information of 30 Carnivora genomes used in this study

Scientific Name	GenBank assembly accession	Assembly version	Sequence technology	Coverage	Contig N50 (bp)
Vulpes lagopus	GCA_004023825.1	VulLag_v1_BIUU	Illumina HiSeq	36.8x	73,976
Lycaon pictus	GCA_001887905.1	LycPicSAfr1.0	Illumina HiSeq	5.7x	113,651
Pteronura brasiliensis	GCA_004024605.1	PteBra_v1_BIUU	Illumina HiSeq	47.7x	90,879
Neovison vison	GCA_900108605.1	NNQGG.v01	Illumina HiSeq	200x	32,336
Gulo gulo	GCA_900006375.2	Gulo_2.2_annotated	Illumina HiSeq	75x	3,657
Mellivora capensis	GCA_004024625.1	MelCap_v1_BIUU	Illumina HiSeq	60x	50,213
Taxidea taxus jeffersonii	GCA_003697995.1	ASM369799v1	Illumina MiSeq	44.0x	37,750
Lutra lutra	GCA_902655055.1	mLutLut1.1	PacBio	63x	30,403,456
Martes zibellina	GCA_012583365.1	ASM1258336v1	Illumina MiSeq	114x	62,196
Ailurus fulgens styani	GCA_002007465.1	ASM200746v1	Illumina HiSeq	115.5x	6,772
Spilogale gracilis	GCA_004023965.1	SpiGra_v1_BIUU	Illumina HiSeq	33.2x	63,054
Arctocephalus gazella	GCA_900500725.1	ArcGazv1.4		200x	538,754
Phoca vitulina	GCA_004348235.1	GSC_HSeal_1.0	Illumina HiSeq	45.0x	283,887
Mirounga angustirostris	GCA_004023865.1	MirAng_v1_BIUU	Illumina HiSeq	35.6x	52,833
Mirounga leonina	GCA_011800145.1	KU_Mleo_1.0	Illumina NovaSeq	100x	197,952
Halichoerus grypus	GCA_012393455.1	Tufts_HGry_1.1	Illumina NextSeq; Oxford Nanopore MinION	39x	1,011,981
Ursus americanus	GCA_003344425.1	ASM334442v1	Illumina; PacBio	100.0x	27,247
Ursus thibetanus	GCA_009660055.1	ASM966005v1	Illumina NovaSeq	90.61x	145,966
Felis nigripes	GCA_004023925.1	FelNig_v1_BIUU	Illumina HiSeq	17.1x	16,007
Panthera onca	GCA_004023805.1	PanOnc_v1_BIUU	Illumina HiSeq	60.7x	62,836
Lynx pardinus	GCA_900661375.1	LYPA1.0		135x	99,542
Prionailurus bengalensis	GCA_005406085.1	Prionailurus_bengalensis_euptilurus_v01	Illumina HiseqX	55X	21,454
Panthera leo	GCA_012313985.1	BGI_AfriLion_1.1	Illumina HiSeq	82x	23,045
Hyaena hyaena	GCA_003009895.1	ASM300989v1	Illumina	56.0x	311,202
Crocuta crocuta	GCA_008692635.1 (latest)	BGI_CrCroc_1.0	Illumina HiSeq	95.2x	97,873
Suricata suricatta	GCA_004023905.1	SurSur_v1_BIUU	Illumina HiSeq	46.9x	148,487
Helogale parvula	GCA_004023845.1	HelPar_v1_BIUU	Illumina HiSeq	32.3x	113,567
Mungos mungo	GCA_004023785.1	MunMun_v1_BIUU	Illumina HiSeq	46.7x	3,832
Cryptoprocta ferox	GCA_004023885.1	CryFer_v1_BIUU	Illumina HiSeq	46.3x	128,639
Paradoxurus hermaphroditus	GCA_004024585.1	ParHer_v1_BIUU	Illumina HiSeq	36.9x	62,870
