# Supplementary material for: FGF gene family characterization provides insights into its adaptive evolution in Carnivora
Source: Ecol Evol. 2021 Jun 29;11(14):9837–47. doi: 10.1002/ece3.7814 (PMC8293770; doi:10.1002/ece3.7814)
Supplement: Supplementary file 4 — Table S2 [file ECE3-11-9837-s002.pdf]

Table S2 Gene accession number for getting the FGF genes in the GeneBank database (FGF1-FGF8)

Scientific name	FGF1	FGF2	FGF3	FGF4	FGF5	FGF6	FGF7	FGF8
Canis lupus familiaris	XM_022408930.1	4	XM_849404.2	XM_540801.4	NM_001048129.1	XM_543862.6	XM_005638175.3	XM_022411595.1
Canis_lupus_dingo	XM_025445541.1	XM_025420484.1	XM_025451678.1	XM_025451762.1	XM_025426711.1	XM_025461876.1	XM_025472855.1	XM_025467769.1
Vulpes vulpes	XM_026018244.1	XM_026016823.1	XM_026003934.1	XM_026003935.1	XM_025998034.1	XM_025995386.1	XM_025998685.1	XM_025989955.1
Enhydra lutris kenyoni	XM_022507360.1	XM_022516534.1	XM_022506580.1	XM_022507758.1	XM_022506211.1	XM_022522476.1	XM_022522715.1	XM_022500036.1
Lontra canadensis	XM_032844238.1	XM_032855740.1	XM_032879917.1	XM_032879083.1	XM_032861323.1	XM_032874367.1	XM_032856547.1	XM_032877440.1
Mustela putorius furo	XM_004744723.2	XM_013053919.1	XM_013059878.1	XM_004759720.2	XM_013047737.1	XM_004778279.1	XM_004751354.2	XM_013054551.1
Mustela erminea	XM_032336323.1	XM_032333350.1	XM_032356261.1	XM_032360238.1	XM_032334704.1	XM_032349070.1	XM_032343679.1	XM_032313008.1
Zalophus californianus	XM_027605139.1	XM_027600087.1	XM_027579617.1	XM_027581513.1	XM_027599529.1	XM_027595716.1	XM_027569745.1	XM_027596557.1
Eumetopias jubatus	XM_028092933.1	XM_028102250.1	XM_028117959.1	XM_028117900.1	XM_028124233.1	XM_028120981.1	XM_028100168.1	XM_028098970.1
Callorhinus ursinus	XM_025852238.1	XM_025874039.1	XM_025878648.1	XM_025879221.1	XM_025887357.1	XM_025851744.1	XM_025883685.1	XM_025880990.1
Odobenus rosmarus divergens	XM_004397733.1	XM_012562786.1	XM_004394042.2	XM_004393799.1	XM_004407220.1	XM_004415979.1	XM_004396538.2	XM_004401945.1
Leptonychotes weddellii		XM_031033416.1	XM_006745271.1	XM_006745270.1	XM_006737422.1	XM_006750023.1	XM_006735611.1	XM_006731097.1
Neomonachus schauinslandi	XM_021701873.1	1	XM_021685281.1	XM_021685155.1	XM_021702408.1	XM_021690739.1	XM_021693812.1	XM_021700023.1
Ursus_arctos_horribilis	XM_026499021.1	XM_026499349.1	XM_026482870.1	XM_026482858.1	XM_026509609.1	XM_026501599.1	XM_026518449.1	XM_026493649.1
Ursus maritimus	XM_008691453.1	XM_008689521.1	XM_008699711.1		XM_008693272.1	XM_008704878.1	XM_008705940.1	XM_008686511.1
Ailuropoda melanoleuca	XM_019795244.1	XM_011226352.2	XM_002927907.1	XM_011235602.1	XM_002912480.3	XM_002920444.3	XM_002917563.3	XM_011236471.1
Panthera_tigris_altaica	XM_007077939.2	1			XM_007074783.1	XM_007089661.1	XM_007081828.2	XM_007080282.1
Puma concolor	XM_025924640.1	1			XM_025921992.1	XM_025930669.1	XM_025918645.1	
Acinonyx jubatus	XM_027042614.1	XM_015074594.2	XM_027045106.1	XM_027045726.1	XM_027059170.1	XM_015061560.2	XM_015063451.2	XM_027062018.1
Panthera pardus	XM_019424552.1	XM_019414908.1	XM_019415372.1	XM_019415371.1	XM_019466439.1	XM_019465313.1	XM_019442165.1	XM_019459679.1
Lynx canadensis	XM_030320477.1	XM_030314481.1	XM_030333859.1	XM_030330834.1	XM_030311921.1	XM_030323346.1	XM_030318226.1	XM_030335224.1
Felis catus	XM_011282701.3	XM_023253395.1	XM_023239755.1	XM_023239032.1	XM_019828138.2	XM_003988285.4	XM_006932534.4	XM_023240312.1
Homo sapiens	NM_000800.5	NM_002006.5	NM_005247.4	NM_002007.4	NM_004464.3	NM_020996.2	NM_002009.4	NM_033163.4
Mus musculus	NM_010197.3	NM_008006.2	NM_008007.2	NM_010202.6	NM_010203.5	NM_010204.1	NM_008008.4	NM_001166361.1
Manis_javanica	XM_017650407.1		XM_017656293.1		XM_017659144.1	XM_017650911.1		XM_017674339.1
Equus caballus	XM_023616891.1	HM769759.1	XM_023654967.1	XM_023654966.1	XM_001492556.5	XM_001494335.4	NM_001163883.1	XM_023629757.1

Table S2 Gene accession number for getting the FGF genes in the GeneBank database (FGF9-FGF17)

Scientific name	FGF9	FGF10	FGF11	FGF12	FGF13	FGF14	FGF16	FGF17
Canis lupus familiaris	XM_844845.5	XM_005619337.3	XM_844648.4	XM_535845.5	XM_022415872.1	XM_003433069.4	XM_549094.5	XM_022409825.1
Canis_lupus_dingo	XM_025462721.1	XM_025434704.1	XM_025428562.1	XM_025425367.1	XM_025460883.1	XM_025441199.1	XM_025466234.1	XM_025463184.1
Vulpes vulpes	XM_025996663.1	XM_026017015.1	XM_026005389.1	XM_026016470.1	XM_026009039.1	XM_026008893.1	XM_025985013.1	XM_026007959.1
Enhydra lutris kenyoni	XM_022522620.1	XM_022493457.1	XM_022524835.1	XM_022507198.1	XM_022521315.1	XM_022502928.1	XM_022524343.1	XM_022518484.1
Lontra canadensis	XM_032838224.1	XM_032851439.1	XM_032839862.1	XM_032875651.1	XM_032841400.1	XM_032881086.1	XM_032840625.1	XM_032855885.1
Mustela putorius furo	XM_004774339.2	XM_004737913.2	XM_004760302.2	XM_004745272.2	XM_004773643.1	XM_004746069.2	XM_004777113.2	XM_004763321.2
Mustela erminea	XM_032315019.1	XM_032337449.1	XM_032321373.1	XM_032337776.1	XM_032329639.1	XM_032315400.1	XM_032331149.1	XM_032332100.1
Zalophus californianus	XM_027587980.1	XM_027607113.1	XM_027568655.1	XM_027587164.1	XM_027609275.1	XM_027591057.1	XM_027608873.1	XM_027599926.1
Eumetopias jubatus	XM_028106545.1	XM_028097923.1	XM_028125139.1	XM_028101058.1	XM_028123839.1	XM_028112062.1	XM_028101429.1	XM_028114420.1
Callorhinus ursinus	XM_025874696.1	XM_025866651.1	XM_025864325.1	XM_025876638.1	XM_025874530.1	XM_025874528.1	XM_025866505.1	XM_025874046.1
Odobenus rosmarus divergens	XM_004401339.2	XM_004415853.2	XM_004398514.2	XM_004391851.1	XM_004405912.1	XM_004404919.1	XM_004415149.1	XM_004402179.1
Leptonychotes weddellii	XM_006727844.1	XM_006729719.1	XM_006738902.1	XM_006733186.1	XM_006742909.2	XM_031031823.1	XM_006726893.1	XM_006744692.1
Neomonachus schauinslandi	XM_021678227.1	XM_021696724.1	XM_021694673.1	XM_021692471.1	XM_021684613.1	XM_021695970.1	XM_021687942.1	XM_021698952.1
Ursus_arctos_horribilis	XM_026492988.1	XM_026506930.1	XM_026520857.1	XM_026496455.1	XM_026479740.1	XM_026493566.1	XM_026480112.1	XM_026519097.1
Ursus maritimus	XM_008692136.1	XM_008692560.1	XM_008688092.1	XM_008705152.1	XM_008701549.1	XM_008684988.1	XM_008704959.1	XM_008702093.1
Ailuropoda melanoleuca	XM_002928925.3	XM_002925486.3	XM_011231359.2	XM_002927247.3	XM_011226385.2	XM_002914849.3	XM_002928760.3	XM_002914781.1
Panthera_tigris_altaica	XM_007074361.2	XM_007095410.2	XM_007091659.2	XM_007099164.2	XM_015544879.1	XM_015544672.1		XM_007093876.1
Puma concolor	XM_025924079.1		XM_025920279.1	XM_025913132.1	XM_025934028.1	XM_025924255.1	XM_025933878.1	XM_025933047.1
Acinonyx jubatus	XM_015077768.2	XM_015063308.2	XM_027047814.1	XM_027061185.1	XM_027054740.1	XM_027065183.1	XM_027053769.1	XM_027077468.1
Panthera pardus	XM_019447989.1	XM_019464312.1	XM_019413597.1	XM_019463518.1	XM_019421519.1	XM_019443950.1	XM_019436172.1	XM_019467034.1
Lynx canadensis		XM_030330230.1	XM_030296850.1		XM_030305582.2	XM_030313640.1	XM_030306344.1	XM_030311860.1
Felis catus	XM_003980257.5	XM_023260856.1	XM_003996171.5	XM_003991788.5	XM_023249008.1	XM_019829208.2	XM_004000660.4	XM_003984716.5
Homo sapiens	NM_002010.3	NM_004465.2	NM_004112.4	NM_021032.4	NM_001139500.2	NM_175929.2	NM_003868.3	NM_003867.4
Mus musculus	NM_013518.4	NM_008002.4	NM_010198.3	NM_183064.5	NM_001290414.1	NM_207667.3	NM_030614.2	NM_008004.5
Manis_javanica	XM_017653637.1	XM_017661370.1	XM_017675204.1	XM_017672438.1	XM_017656461.1	XM_017676071.1	XM_017669001.1	XM_017660651.1
Equus caballus	XM_005601094.3	XM_001498109.4	XM_001918148.4	XM_001500259.6	XM_023634065.1	XM_023621799.1	XM_023634057.1	XM_023636127.1

Table S2 Gene accession number for getting the FGF genes in the GeneBank database (FGF18-FGF23)

Scientific name	FGF18	FGF19	FGF20	FGF21	FGF22	FGF23
Canis lupus familiaris	XM_849332.5	XM_540802.4	XM_005630043.2	XM_022424196.1	XM_005633169.3	XM_849487.2
Canis_lupus_dingo	XM_025434836.1	XM_025451677.1	XM_025472223.1	XM_025424370.1	XM_025456632.1	XM_025461875.1
Vulpes vulpes	XM_026010354.1	XM_026003936.1	XM_025984568.1	XM_026013782.1		XM_025995571.1
Enhydra lutris kenyoni	XM_022493628.1	XM_022506446.1	XM_022499013.1	XM_022523709.1	XM_022525391.1	XM_022522369.1
Lontra canadensis	XM_032851799.1	XM_032879082.1	XM_032844942.1	XM_032839271.1	XM_032847057.1	XM_032874836.1
Mustela putorius furo	XM_004737611.1	XM_004759618.2	XM_004777456.2	XM_004767394.1	XM_013048698.1	XM_004778280.2
Mustela erminea	XM_032337021.1	XM_032358766.1	XM_032329331.1	XM_032324272.1	XM_032304228.1	XM_032349071.1
Zalophus californianus	XM_027606211.1		XM_027600277.1	XM_027620341.1	XM_027583456.1	XM_027594475.1
Eumetopias jubatus	XM_028102794.1		XM_028099680.1	XM_028091380.1		XM_028120978.1
Callorhinus ursinus	XM_025866622.1	XM_025878664.1	XM_025885887.1	XM_025849424.1	XM_025861064.1	XM_025851732.1
Odobenus rosmarus divergens	XM_004392007.1	XM_012567902.1	XM_004409519.1	XM_004415096.2	XM_004395444.1	XM_004415978.1
Leptonychotes weddellii	XM_031030350.1	XM_006744527.2	XM_006727391.1	XM_006747529.1	XM_031022408.1	XM_006750021.1
Neomonachus schauinslandi	XM_021698322.1	XM_021685280.1	XM_021694262.1	XM_021681243.1	XM_021705414.1	XM_021690705.1
Ursus_arctos_horribilis	XM_026510962.1	XM_026482872.1	XM_026508278.1	XM_026481711.1	XM_026480834.1	XM_026502646.1
Ursus maritimus	XM_008692708.1	1	XM_008710935.1	XM_008684516.1		XM_008704877.1
Ailuropoda melanoleuca	XM_011229459.2	1	XM_002929386.3	XM_002917864.3	3	XM_002920450.3
Panthera_tigris_altaica	XM_007076813.1	XM_007094131.1	XM_007099018.1	XM_015539757.1	XM_007075729.2	XM_007089695.1
Puma concolor			XM_025932972.1	XM_025914766.1		XM_025930938.1
Acinonyx jubatus	XM_015070501.2		XM_027077487.1	XM_015087189.2	XM_027049506.1	XM_015061533.2
Panthera pardus	XM_019450298.1	XM_019415262.1	XM_019467062.1	XM_019425777.1	XM_019433935.1	XM_019464807.1
Lynx canadensis	XM_030305367.2	XM_030333858.1	XM_030311850.1	XM_030298966.1	XM_030297871.1	XM_030321784.1
Felis catus	XM_011285502.3	XM_003993764.2	XM_023252220.1	XM_003997528.3	XM_023242667.1	XM_011283757.3
Homo sapiens	NM_003862.3	NM_005117.3	NM_019851.3	NM_019113.4	NM_020637.1	NM_020638.3
Mus musculus	NM_008005.2	NM_008003.2	NM_030610.2	NM_020013.4	NM_023304.2	NM_022657.4
Manis_javanica	XM_017640749.1		XM_017653982.1	XM_017665663.1	XM_017644545.1	XM_017650910.1
Equus caballus	XM_023617246.1	XM_023654623.1	XM_001488229.6	XM_001489152.3	XM_023644270.1	XM_001491419.3
