# Supplementary material for: FGF gene family characterization provides insights into its adaptive evolution in Carnivora
Source: Ecol Evol. 2021 Jun 29;11(14):9837–47. doi: 10.1002/ece3.7814 (PMC8293770; doi:10.1002/ece3.7814)
Supplement: Supplementary file 5 — Table S3 [file ECE3-11-9837-s007.pdf]

Table S3 Species used for selection analysis (FGF1-7)

Scientific Name	Abbreviation	FGF1	FGF2	FGF3	FGF4	FGF5	FGF6	FGF7
Canis lupus familiaris	Cfam	XM_022408930.1		XM_849404.2	XM_540801.4	NM_001048129.1	XM_543862.6	XM_005638175.3
Canis_lupus_dingo	Cdin	XM_025445541.1	XM_025420484.1	XM_025451678.1	XM_025451762.1	XM_025426711.1	XM_025461876.1	XM_025472855.1
Vulpes vulpes	Vvul	XM_026018244.1	XM_026016823.1	XM_026003934.1	XM_026003935.1	XM_025998034.1	XM_025995386.1	XM_025998685.1
Vulpes lagopus	Vlag	√	√	√	√	√	√	√
Lycaon pictus	Lpic	√	√	√	√	√	√	√
Enhydra lutris kenyoni	Eken	XM_022507360.1	XM_022516534.1	XM_022506580.1	XM_022507758.1	XM_022506211.1	XM_022522476.1	XM_022522715.1
Pteronura brasiliensis	Pbra	√		√	√		√	√
Lutra lutra	Llut	√	√	√	√	√	√	√
Lontra canadensis	Lcan	XM_032844238.1	XM_032855740.1	XM_032879917.1	XM_032879083.1	XM_032861323.1	XM_032874367.1	XM_032856547.1
Mustela putorius furo	Mfur	XM_004744723.2	XM_013053919.1	XM_013059878.1	XM_004759720.2	XM_013047737.1	XM_004778279.1	XM_004751354.2
Neovison vison	Nvis	√	√	√	√	√	√	√
Mustela erminea	Merm	XM_032336323.1	XM_032333350.1	XM_032356261.1	XM_032360238.1	XM_032334704.1	XM_032349070.1	XM_032343679.1
Martes zibellina	Mzib	√	√	√	√	√	√	√
Gulo gulo	Ggul	√	√	√		√	√	√
Mellivora capensis	Mcap	√		√	√	√	√	√
Taxidea taxus jeffersonii	Tjef	√	√	√	√		√	√
Ailurus fulgens styani	Asty	√				√	√	√
Spilogale gracilis	Sgra	√		√	√	√	√	√
Zalophus californianus	Zcal	XM_027605139.1	XM_027600087.1	XM_027579617.1	XM_027581513.1	XM_027599529.1	XM_027595716.1	XM_027569745.1
Eumetopias jubatus	Ejub	XM_028092933.1	XM_028102250.1	XM_028117959.1	XM_028117900.1	XM_028124233.1	XM_028120981.1	XM_028100168.1
Callorhinus ursinus	Curs	XM_025852238.1	XM_025874039.1	XM_025878648.1	XM_025879221.1	XM_025887357.1	XM_025851744.1	XM_025883685.1
Arctocephalus gazella	Agaz	√			√		√	
Odobenus rosmarus divergens	Odiv	XM_004397733.1	XM_012562786.1	XM_004394042.2	XM_004393799.1	XM_004407220.1	XM_004415979.1	XM_004396538.2
Leptonychotes weddellii	Lwed	√	XM_031033416.1			XM_006737422.1	XM_006750023.1	XM_006735611.1
Neomonachus schauinslandi	Msch	XM_021701873.1	XM_021681500.1	XM_021685281.1	XM_021685155.1	XM_021702408.1	XM_021690739.1	XM_021693812.1
Phoca vitulina	Pvit	√	√	√	√	√	√	√
Mirounga angustirostris	Mang	√	√	√	√	√	√	√
Mirounga leonina	Mleo	√	√	√	√	√	√	√
Halichoerus grypus	Hgry	√	√	√	√		√	√
Ursus_arctos_horribilis	Uhor	XM_026499021.1		XM_026482870.1	XM_026482858.1	XM_026509609.1	XM_026501599.1	XM_026518449.1

Ursus maritimus	Umar	XM_008691453.1	XM_008689521.1	XM_008699711.1		XM_008693272.1	XM_008704878.1	XM_008705940.1
Ursus americanus	Uame	√	√	√		√	√	√
Ursus thibetanus	Uthi	√		√	√	√	√	√
Ailuropoda melanoleuca	Amel	XM_019795244.1	XM_011226352.2	XM_002927907.1	XM_011235602.1	XM_002912480.3	XM_002920444.3	XM_002917563.3
Panthera_tigris_altaica	Palt	XM_007077939.2				XM_007074783.1	XM_007089661.1	XM_007081828.2
Puma concolor	Pcon	XM_025924640.1	XM_025922196.1			XM_025921992.1	XM_025930669.1	XM_025918645.1
Panthera leo	Pleo	√			√	√	√	√
Panthera onca	Ponc	√	√	√	√	√	√	√
Acinonyx jubatus	Ajub	XM_027042614.1	XM_015074594.2	XM_027045106.1	XM_027045726.1	XM_027059170.1	XM_015061560.2	XM_015063451.2
Panthera pardus	Ppar	XM_019424552.1	XM_019414908.1	XM_019415372.1	XM_019415371.1	XM_019466439.1	XM_019465313.1	XM_019442165.1
Lynx pardinus	Lpar	√			√		√	√
Lynx canadensis	Lycan	XM_030320477.1	XM_030314481.1	XM_030333859.1	XM_030330834.1	XM_030311921.1	XM_030323346.1	XM_030318226.1
Felis catus	Fcat	XM_011282701.3	XM_023253395.1	XM_023239755.1	XM_023239032.1	XM_019828138.2	XM_003988285.4	XM_006932534.4
Felis nigripes	Fnig	√			√		√	√
Prionailurus bengalensis	Pben	√		√	√	√	√	√
Hyaena hyaena	Hhya	√	√	√	√	√	√	√
Crocuta crocuta	Ccro	√				√	√	√
Suricata suricatta	Ssur	√	√	√	√	√	√	√
Helogale parvula	Hpar	√	√	√	√		√	√
Mungos mungo	Mmun	√	√	√	√	√	√	√
Cryptoprocta ferox	Cfer	√	√		√		√	√
Paradoxurus hermaphroditus	Pher	√	√		√		√	√
Homo sapiens	Hsap	NM_000800.5	NM_002006.5	NM_005247.4	NM_002007.4	NM_004464.3	NM_020996.2	NM_002009.4
Mus musculus	Mmus	NM_010197.3	NM_008006.2	NM_008007.2	NM_010202.6	NM_010203.5	NM_010204.1	NM_008008.4
Manis_javanica	Mjav	XM_017650407.1				XM_017659144.1	XM_017650911.1	
Equus caballus	Ecab	XM_023616891.1	HM769759.1	XM_023654967.1	XM_023654966.1	XM_001492556.5	XM_001494335.4	NM_001163883.1

notes:The " √ " means the genes were newly identified in this study, and the gene accession number means the genes were downloaded from GeneBank Database, others means that the genes were omitted in this analysis.

Table S3 Species used for selection analysis (FGF8-FGF14)

Scientific Name	Abbreviation	FGF8	FGF9	FGF10	FGF11	FGF12	FGF13	FGF14
Canis lupus familiaris	Cfam	XM_022411595.1	XM_844845.5	XM_005619337.3	XM_844648.4	XM_535845.5	XM_022415872.1	XM_003433069.4
Canis_lupus_dingo	Cdin	XM_025467769.1	XM_025462721.1	XM_025434704.1	XM_025428562.1	XM_025425367.1	XM_025460883.1	XM_025441199.1
Vulpes vulpes	Vvul	XM_025989955.1	XM_025996663.1	XM_026017015.1	XM_026005389.1	XM_026016470.1	XM_026009039.1	XM_026008893.1
Vulpes lagopus	Vlag		√	√	√	√	√	√
Lycaon pictus	Lpic		√	√	√	√	√	√
Enhydra lutris kenyoni	Eken	XM_022500036.1	XM_022522620.1	XM_022493457.1	XM_022524835.1	XM_022507198.1	XM_022521315.1	XM_022502928.1
Pteronura brasiliensis	Pbra	√	√	√	√	√	√	√
Lutra lutra	Llut		√	√	√	√	√	√
Lontra canadensis	Lcan	XM_032877440.1	XM_032838224.1	XM_032851439.1	XM_032839862.1	XM_032875651.1	XM_032841400.1	XM_032881086.1
Mustela putorius furo	Mfur	XM_013054551.1	XM_004774339.2	XM_004737913.2	XM_004760302.2	XM_004745272.2	XM_004773643.1	XM_004746069.2
Neovison vison	Nvis	√	√	√	√	√	√	√
Mustela erminea	Merm	XM_032313008.1	XM_032315019.1	XM_032337449.1	XM_032321373.1	XM_032337776.1	XM_032329639.1	XM_032315400.1
Martes zibellina	Mzib	√	√	√	√	√	√	√
Gulo gulo	Ggul	√	√	√	√	√	√	
Mellivora capensis	Mcap	√	√	√	√	√	√	√
Taxidea taxus jeffersonii	Tjef	√	√	√	√	√		√
Ailurus fulgens styani	Asty		√	√		√	√	√
Spilogale gracilis	Sgra	√	√	√	√			√
Zalophus californianus	Zcal	XM_027596557.1	XM_027587980.1	XM_027607113.1	XM_027568655.1	XM_027587164.1	XM_027609275.1	XM_027591057.1
Eumetopias jubatus	Ejub	XM_028098970.1	XM_028106545.1	XM_028097923.1	XM_028125139.1	XM_028101058.1	XM_028123839.1	XM_028112062.1
Callorhinus ursinus	Curs	XM_025880990.1	XM_025874696.1	XM_025866651.1	XM_025864325.1	XM_025876638.1	XM_025874530.1	XM_025874528.1
Arctocephalus gazella	Agaz		√	√			√	√
Odobenus rosmarus divergens	Odiv	XM_004401945.1	XM_004401339.2	XM_004415853.2	XM_004398514.2	XM_004391851.1	XM_004405912.1	XM_004404919.1
Leptonychotes weddellii	Lwed	XM_006731097.1	XM_006727844.1	XM_006729719.1	XM_006738902.1	XM_006733186.1	XM_006742909.2	XM_031031823.1
Neomonachus schauinslandi	Msch	XM_021700023.1	XM_021678227.1	XM_021696724.1	XM_021694673.1	XM_021692471.1	XM_021684613.1	XM_021695970.1
Phoca vitulina	Pvit	√	√	√			√	√
Mirounga angustirostris	Mang		√	√	√	√	√	√
Mirounga leonina	Mleo	√	√	√	√	√	√	√
Halichoerus grypus	Hgry		√	√	√			√

Ursus_arctos_horribilis	Uhor	XM_026493649.1	XM_026492988.1	XM_026506930.1	XM_026520857.1	XM_026496455.1	XM_026479740.1	XM_026493566.1
Ursus maritimus	Umar	XM_008686511.1	XM_008692136.1	XM_008692560.1	XM_008688092.1	XM_008705152.1	XM_008701549.1	XM_008684988.1
Ursus americanus	Uame	√	√	√	√	√	√	√
Ursus thibetanus	Uthi	√	√	√	√	√	√	√
Ailuropoda melanoleuca	Amel	XM_011236471.1	XM_002928925.3	XM_002925486.3	XM_011231359.2	XM_002927247.3	XM_011226385.2	XM_002914849.3
Panthera_tigris_altaica	Palt	XM_007080282.1	XM_007074361.2	XM_007095410.2	XM_007091659.2	XM_007099164.2	XM_015544879.1	XM_015544672.1
Puma concolor	Pcon		XM_025924079.1		XM_025920279.1	XM_025913132.1	XM_025934028.1	XM_025924255.1
Panthera leo	Pleo	√	√	√	√	√	√	√
Panthera onca	Ponc	√	√	√	√	√	√	√
Acinonyx jubatus	Ajub	XM_027062018.1	XM_015077768.2	XM_015063308.2	XM_027047814.1	XM_027061185.1	XM_027054740.1	XM_027065183.1
Panthera pardus	Ppar	XM_019459679.1	XM_019447989.1	XM_019464312.1	XM_019413597.1	XM_019463518.1	XM_019421519.1	XM_019443950.1
Lynx pardinus	Lpar		√	√	√	√	√	√
Lynx canadensis	Lycan	XM_030335224.1		XM_030330230.1	XM_030296850.1	√	XM_030305582.2	XM_030313640.1
Felis catus	Fcat	XM_023240312.1	XM_003980257.5	XM_023260856.1	XM_003996171.5	XM_003991788.5	XM_023249008.1	XM_019829208.2
Felis nigripes	Fnig	√	√		√	√		
Prionailurus bengalensis	Pben	√	√	√	√	√	√	√
Hyaena hyaena	Hhya	√	√	√	√	√	√	√
Crocuta crocuta	Ccro		√	√	√	√		√
Suricata suricatta	Ssur	√	√	√	√	√	√	√
Helogale parvula	Hpar	√	√	√	√	√	√	√
Mungos mungo	Mmun	√	√	√	√	√	√	√
Cryptoprocta ferox	Cfer	√	√	√	√	√	√	√
Paradoxurus hermaphroditus	Pher	√	√	√	√		√	√
Homo sapiens	Hsap	NM_033163.4	NM_002010.3	NM_004465.2	NM_004112.4	NM_021032.4	NM_001139500.2	NM_175929.2
Mus musculus	Mmus	NM_001166361.1	NM_013518.4	NM_008002.4	NM_010198.3	NM_183064.5	NM_001290414.1	NM_207667.3
Manis_javanica	Mjav	XM_017674339.1	XM_017653637.1	XM_017661370.1	XM_017675204.1	XM_017672438.1	XM_017656461.1	XM_017676071.1
Equus caballus	Ecab	XM_023629757.1	XM_005601094.3	XM_001498109.4	XM_001918148.4	XM_001500259.6	XM_023634065.1	XM_023621799.1

notes:The " √ " means the genes were newly identified in this study, and the gene accession number means the genes were downloaded from GeneBank Database, others means that the genes were omitted in this analysis.

Table S3 Species used for selection analysis (FGF16-FGF22)

Scientific Name	Abbreviation	FGF16	FGF17	FGF18	FGF19	FGF20	FGF21	FGF22
Canis lupus familiaris	Cfam	XM_549094.5	XM_022409825.1	XM_849332.5		XM_005630043.2	XM_022424196.1	XM_005633169.3
Canis_lupus_dingo	Cdin	XM_025466234.1	XM_025463184.1	XM_025434836.1	XM_025451677.1	XM_025472223.1	XM_025424370.1	XM_025456632.1
Vulpes vulpes	Vvul	XM_025985013.1	XM_026007959.1	XM_026010354.1	XM_026003936.1	XM_025984568.1	XM_026013782.1	
Vulpes lagopus	Vlag	√	√		√	√	√	√
Lycaon pictus	Lpic		√	√			√	√
Enhydra lutris kenyoni	Eken	XM_022524343.1	XM_022518484.1	XM_022493628.1	XM_022506446.1	XM_022499013.1	XM_022523709.1	XM_022525391.1
Pteronura brasiliensis	Pbra	√		√	√	√	√	√
Lutra lutra	Llut	√	√	√	√	√	√	√
Lontra canadensis	Lcan	XM_032840625.1	XM_032855885.1	XM_032851799.1	XM_032879082.1	XM_032844942.1	XM_032839271.1	XM_032847057.1
Mustela putorius furo	Mfur	XM_004777113.2	XM_004763321.2	XM_004737611.1	XM_004759618.2	XM_004777456.2	XM_004767394.1	XM_013048698.1
Neovison vison	Nvis	√	√	√	√	√	√	√
Mustela erminea	Merm	XM_032331149.1	XM_032332100.1	XM_032337021.1	XM_032358766.1	XM_032329331.1	XM_032324272.1	XM_032304228.1
Martes zibellina	Mzib	√	√	√	√	√	√	
Gulo gulo	Ggul	√		√	√	√	√	
Mellivora capensis	Mcap	√	√	√	√	√	√	√
Taxidea taxus jeffersonii	Tjef	√			√	√	√	√
Ailurus fulgens styani	Asty	√		√	√	√	√	
Spilogale gracilis	Sgra	√			√	√	√	√
Zalophus californianus	Zcal	XM_027608873.1	XM_027599926.1	XM_027606211.1		XM_027600277.1	XM_027620341.1	XM_027583456.1
Eumetopias jubatus	Ejub	XM_028101429.1	XM_028114420.1	XM_028102794.1		XM_028099680.1	XM_028091380.1	
Callorhinus ursinus	Curs	XM_025866505.1	XM_025874046.1	XM_025866622.1	XM_025878664.1	XM_025885887.1	XM_025849424.1	XM_025861064.1
Arctocephalus gazella	Agaz	√	√	√			√	√
Odobenus rosmarus divergens	Odiv	XM_004415149.1	XM_004402179.1	XM_004392007.1	XM_012567902.1	XM_004409519.1	XM_004415096.2	XM_004395444.1
Leptonychotes weddellii	Lwed	XM_006726893.1	XM_006744692.1	XM_031030350.1	XM_006744527.2	XM_006727391.1	XM_006747529.1	XM_031022408.1
Neomonachus schauinslandi	Msch	XM_021687942.1	XM_021698952.1	XM_021698322.1	XM_021685280.1	XM_021694262.1	XM_021681243.1	XM_021705414.1
Phoca vitulina	Pvit	√	√	√	√	√	√	√
Mirounga angustirostris	Mang	√	√	√	√	√	√	√
Mirounga leonina	Mleo	√	√	√	√	√	√	√

Halichoerus grypus	Hgry	√	√	√	√	√	√	√
Ursus_arctos_horribilis	Uhor	XM_026480112.1	XM_026519097.1	XM_026510962.1	XM_026482872.1	XM_026508278.1	XM_026481711.1	XM_026480834.1
Ursus maritimus	Umar	XM_008704959.1	XM_008702093.1	XM_008692708.1		XM_008710935.1	XM_008684516.1	
Ursus americanus	Uame	√	√	√	√	√	√	√
Ursus thibetanus	Uthi	√	√	√	√	√	√	√
Ailuropoda melanoleuca	Amel	XM_002928760.3	XM_002914781.1	XM_011229459.2		XM_002929386.3	XM_002917864.3	
Panthera_tigris_altaica	Palt			XM_007076813.1	XM_007094131.1	XM_007099018.1	XM_015539757.1	XM_007075729.2
Puma concolor	Pcon	XM_025933878.1	XM_025933047.1			XM_025932972.1	XM_025914766.1	
Panthera leo	Pleo	√	√	√	√	√	√	√
Panthera onca	Ponc	√	√	√	√	√	√	√
Acinonyx jubatus	Ajub	XM_027053769.1	XM_027077468.1	XM_015070501.2	√	XM_027077487.1	XM_015087189.2	XM_027049506.1
Panthera pardus	Ppar	XM_019436172.1	XM_019467034.1	XM_019450298.1	XM_019415262.1	XM_019467062.1	XM_019425777.1	XM_019433935.1
Lynx pardinus	Lpar		√	√	√	√	√	
Lynx canadensis	Lycan	XM_030306344.1	XM_030311860.1	XM_030305367.2	XM_030333858.1	XM_030311850.1	XM_030298966.1	XM_030297871.1
Felis catus	Fcat	XM_004000660.4	XM_003984716.5	XM_011285502.3	XM_003993764.2	XM_023252220.1	XM_003997528.3	XM_023242667.1
Felis nigripes	Fnig		√	√		√	√	√
Prionailurus bengalensis	Pben	√	√	√	√	√	√	√
Hyaena hyaena	Hhya	√	√	√	√	√	√	√
Crocuta crocuta	Ccro		√	√			√	
Suricata suricatta	Ssur	√	√	√	√	√		√
Helogale parvula	Hpar	√	√	√	√	√	√	√
Mungos mungo	Mmun	√	√	√	√	√		√
Cryptoprocta ferox	Cfer	√	√	√	√	√	√	√
Paradoxurus hermaphroditus	Pher	√	√	√	√	√		√
Homo sapiens	Hsap	NM_003868.3	NM_003867.4	NM_003862.3	NM_005117.3	NM_019851.3	NM_019113.4	NM_020637.1
Mus musculus	Mmus	NM_030614.2	NM_008004.5	NM_008005.2	NM_008003.2(FGF15)	NM_030610.2	NM_020013.4	NM_023304.2
Manis_javanica	Mjav	XM_017669001.1	XM_017660651.1	XM_017640749.1		XM_017653982.1	XM_017665663.1	XM_017644545.1
Equus caballus	Ecab	XM_023634057.1	XM_023636127.1	XM_023617246.1	XM_023654623.1	XM_001488229.6	XM_001489152.3	XM_023644270.1

notes:The " √ " means the genes were newly identified in this study, and the gene accession number means the genes were downloaded from GeneBank Database, others means that the genes were omitted in this analysis.

Table S3 Species used for selection analysis (FGF23)

Scientific Name	Abbreviation	FGF23
Canis lupus familiaris	Cfam	XM_849487.2
Canis_lupus_dingo	Cdin	XM_025461875.1
Vulpes vulpes	Vvul	XM_025995571.1
Vulpes lagopus	Vlag	
Lycaon pictus	Lpic	
Enhydra lutris kenyoni	Eken	XM_022522369.1
Pteronura brasiliensis	Pbra	
Lutra lutra	Llut	√
Lontra canadensis	Lcan	XM_032874836.1
Mustela putorius furo	Mfur	XM_004778280.2
Neovison vison	Nvis	√
Mustela erminea	Merm	XM_032349071.1
Martes zibellina	Mzib	√
Gulo gulo	Ggul	√
Mellivora capensis	Mcap	
Taxidea taxus jeffersonii	Tjef	√
Ailurus fulgens styani	Asty	
Spilogale gracilis	Sgra	√
Zalophus californianus	Zcal	XM_027594475.1
Eumetopias jubatus	Ejub	XM_028120978.1
Callorhinus ursinus	Curs	XM_025851732.1
Arctocephalus gazella	Agaz	√
Odobenus rosmarus divergens	Odiv	XM_004415978.1
Leptonychotes weddellii	Lwed	XM_006750021.1
Neomonachus schauinslandi	Msch	XM_021690705.1
Phoca vitulina	Pvit	√
Mirounga angustirostris	Mang	√
Mirounga leonina	Mleo	√
Halichoerus grypus	Hgry	√

Ursus_arctos_horribilis	Uhor	XM_026502646.1
Ursus maritimus	Umar	XM_008704877.1
Ursus americanus	Uame	√
Ursus thibetanus	Uthi	√
Ailuropoda melanoleuca	Amel	XM_002920450.3
Panthera_tigris_altaica	Palt	XM_007089695.1
Puma concolor	Pcon	XM_025930938.1
Panthera leo	Pleo	√
Panthera onca	Ponc	√
Acinonyx jubatus	Ajub	XM_015061533.2
Panthera pardus	Ppar	XM_019464807.1
Lynx pardinus	Lpar	√
Lynx canadensis	Lycan	XM_030321784.1
Felis catus	Fcat	XM_011283757.3
Felis nigripes	Fnig	
Prionailurus bengalensis	Pben	√
Hyaena hyaena	Hhya	√
Crocuta crocuta	Ccro	√
Suricata suricatta	Ssur	√
Helogale parvula	Hpar	
Mungos mungo	Mmun	√
Cryptoprocta ferox	Cfer	√
Paradoxurus hermaphroditus	Pher	√
Homo sapiens	Hsap	NM_020638.3
Mus musculus	Mmus	NM_022657.4
Manis_javanica	Mjav	XM_017650910.1
Equus caballus	Ecab	XM_001491419.3

notes:The " √ " means the genes were newly identified in this study, and the gene accession number means the genes were downloaded from GeneBank Database, others means that the genes were omitted in this analysis.
