# Supplementary material for: FGF gene family characterization provides insights into its adaptive evolution in Carnivora
Source: Ecol Evol. 2021 Jun 29;11(14):9837–47. doi: 10.1002/ece3.7814 (PMC8293770; doi:10.1002/ece3.7814)
Supplement: Supplementary file 6 — Table S4 [file ECE3-11-9837-s001.pdf]

Table S4 The ω (dN/dS) values from the free ratio model which allows all branched have different ω. (FGF1-FGF10)

Species	FGF1ω	Species	FGF3ω	Species	FGF4ω	Species	FGF5ω	Species	FGF6ω	Species	FGF7ω	Species	FGF9ω	Species	FGF10ω
Cfer	0.5036	Mmun	0.3377	Cfer	0.0131	Mfur	0.639	Cfer	0.1761	Pher	0.1117	Pbra	0.341	Mmun	0.7121
Pher	0.0975	Ssur	0.0311	Ssur	0.1005	Mzib	0.4951	Pher	0.0694	Mzib	0.3359	Llut	0.341	Hpar	0.3545
Tjef	0.1272	Hhya	0.0311	Hhya	0.0269	Mcap	1.539	Ppar	0.3643	Sgra	0.0802	Asty	0.1691	Vvul	0.3476
Pbra	0.7885	Pben	0.095	Pher	0.0723	Sgra	1.5565	Tjef	0.7411	Ecab	0.0258	Sgra	0.0405	Lcan	0.3532
Lcan	0.2582	Vlag	0.0385	Fnig	0.1114	Mjav	0.2672	Pbra	0.2393	Mmus	0.0752	Mjav	0.0288	Mcap	0.3545
Mcap	0.2497	Tjef	0.0601	Nvis	0.25	Ecab	0.2241	Llut	0.3648	Hsap	0.1515			Asty	0.0578
Sgra	0.2602	Pbra	0.0334	Mzib	0.0145	Mmus	0.7607	Lcan	0.3655					Sgra	0.0691
Mjav	0.023	Mfur	0.0186	Sgra	0.0292	Hsap	0.3935	Nvis	0.7423					Agaz	0.1759
Mmus	0.0768	Sgra	0.0325	Ecab	0.0503			Mcap	0.1205					Mjav	0.0403
		Curs	0.1329	Mmus	0.026			Sgra	1.0765					Ecab	0.0478
		Uthi	0.1262	Hsap	0.0339			Amel	0.2905					Mmus	0.1127
		Umar	0.0937					Mjav	0.2332						
		Ecab	0.0311					Ecab	0.0642						
		Mmus	0.0326					Mmus	0.0433						
		Hsap	0.0808					Hsap	0.2683						

Notes: The ω collected in this table were that both the dN and dS were no less than 0.0002. The species that marked in red were semiaquatic Carnivore

Table S4 The ω (dN/dS) values from the free ratio model which allows all branched have different ω.(FGF11-GF19)

Species	FGF11ω	Species	FGF13ω	Species	FGF14ω	Species	FGF16ω	Species	FGF17ω	Species	FGF18ω	Species	FGF19ω
Ssur	0.0982	Hhya	0.2886	Lpic	0.1192	Cfer	0.8167	Ssur	0.1108	Lpic	0.1815	Cfer	0.1685
Hhya	0.2768	Pher	0.0354	Asty	0.0715	Hhya	0.0493	Uthi	0.2011	Mfur	0.1761	Mmun	0.1206
Uame	0.2935	Hsap	0.0332	Mjav	0.0814	Pher	0.1355	Ecab	0.0266	Lwed	0.2606	Ssur	0.2379
Mjav	0.0858			Hsap	0.093	Sgra	0.0462	Mmus	0.0077	Mjav	0.0434	Hhya	0.1652
Ecab	0.0738					Mjav	0.0442			Hsap	0.021	Pher	0.1732
Mmus	0.0324					Hsap	0.0454					Cdin	0.2545
Hsap	0.1256											Pbra	0.4152
												Llut	0.421
												Eken	0.2772
												Nvis	0.505
												Merm	0.281
												Mzib	0.0871
												Mcap	0.6721
												Asty	0.1008
												Sgra	0.09
												Odiv	0.128
												Ecab	0.081
												Mmus	0.2462
												Hsap	0.1277

Notes: The ω collected in this table were that both the dN and dS were no less than 0.0002. The species that marked in red were semiaquatic Carnivore

Table S4 The ω (dN/dS) values from the free ratio model which allows all branched have different ω (FGF20-FGF23).

Species	FGF20ω	Species	FGF21ω	Species	FGF22ω	Species	FGF23ω
Fnig	0.3415	Cfer	0.1687	Pher	0.0529	Cfer	0.1071
Cfam	16.271	Hpar	0.3632	Pbra	0.1053	Ssur	0.1435
Sgra	0.0533	Ccro	0.2282	Lcan	0.0952	Vvul	0.052
Odiv	0.3312	Hhya	0.1696	Sgra	0.0129	Tjef	0.1182
Mjav	0.0587	Palt	0.5524	Zcal	0.0443	Llut	0.218
Ecab	0.036	Pben	0.0882	Mjav	0.0028	Sgra	0.2046
		Ajub	1.0719	Mmus	0.0182	Mjav	0.05
		Tjef	0.1496			Ecab	0.0457
		Pbra	0.1428			Mmus	0.1167
		Llut	2.96			Hsap	0.0824
		Eken	1.2663				
		Lcan	0.3719				
		Nvis	0.1769				
		Mfur	0.7468				
		Mcap	0.1963				
		Asty	0.4067				
		Sgra	0.0808				
		Lwed	0.6017				
		Mleo	0.3675				
		Msch	0.2482				
		Mjav	0.3				
		Ecab	0.256				
		Mmus	0.171				
		Hsap	0.3765				

Notes: The ω collected in this table were that both the dN and dS were no less than 0.0002. The species that marked in red were semiaquatic Carnivore
