# Supplementary material for: FGF gene family characterization provides insights into its adaptive evolution in Carnivora
Source: Ecol Evol. 2021 Jun 29;11(14):9837–47. doi: 10.1002/ece3.7814 (PMC8293770; doi:10.1002/ece3.7814)
Supplement: Supplementary file 7 — Table S5 [file ECE3-11-9837-s004.pdf]

Table S5 The comparison of two ratio model and one ratio model

gene	Two ratio model			One ratio model		P value
	Terrestrial dN/dS	Semiaquatic dN/dS	lnL	dN/dS	lnL	
FGF1	0.0857	0.3356	-1114.112465	0.1031	-1117.221211	0.01264942
FGF2	0.0272	NA	-452.342581	0.0238	-452.712617	0.38963781
FGF3	0.044	0.0701	-1757.429691	0.0476	-1758.183747	0.2194274
FGF4	0.0269	0.0311	-2010.268762	0.0272	-2010.310426	0.77283706
FGF5	0.5859	0.3638	-1539.105506	0.5646	-1539.432678	0.41856385
FGF6	0.1607	0.623	-1824.569691	0.1914	-1829.801216	0.00121786
FGF7	0.072	0.0325	-1591.50934	0.0688	-1591.842054	0.41465017
FGF8	0.0041	NA	-488.950445	0.0036	-489.066702	0.62966597
FGF9	0.0234	0.0485	-1221.60166	0.0259	-1221.937632	0.41237466
FGF10	0.0595	0.2106	-1598.398647	0.0743	-1601.603327	0.01135203
FGF11	0.0456	0.0352	-1267.702916	0.0436	-1265.727537	0.04685026
FGF12	0.0088	NA	-1395.669791	0.0079	-1395.962891	0.44389233
FGF13	0.0135	NA	-1810.512851	0.0123	-1811.241857	0.2272467
FGF14	0.0352	0.0259	-1453.016111	0.0342	-1453.059534	0.76822577
FGF16	0.0528	NA	-1064.940895	0.0475	-1066.122003	0.1243055
FGF17	0.0072	0.0092	-1658.905032	0.0074	-1658.928905	0.82703287
FGF18	0.0117	0.0809	-1038.071361	0.0176	-1042.410911	0.0032188
FGF19	0.1197	0.2305	-2502.791618	0.1323	-2504.842288	0.04284924
FGF20	0.0314	0.0436	-1323.185462	0.0326	-1323.263061	0.69361664
FGF21	0.2039	0.4575	-4179.9515	0.2364	-4187.195246	0.00014109
FGF22	0.0529	0.0998	-1000.801951	0.0581	-1001.37586	0.28400638
FGF23	0.0754	0.0486	-1218.71524	0.0727	-1218.948269	0.49480628

Notes:The two ratio model allows different dN/dS ratios between Terrestrial branches and Semiaquatic branches, the one ratio model allows only one dN/dS ratio across the carnivora phylogenetic tree, p values were calculated from log likelihood ratio test. The dN/dS ratios that less than 0.0002 were omitted and marked as NA.
